# *Cydonia oblonga* M., A Medicinal Plant Rich in Phytonutrients for Pharmaceuticals

**DOI:** 10.3389/fphar.2016.00163

**Published:** 2016-06-21

**Authors:** Muhammad U. Ashraf, Gulzar Muhammad, Muhammad A. Hussain, Syed N. A. Bukhari

**Affiliations:** ^1^Faculty of Pharmacy, University of SargodhaSargodha, Pakistan; ^2^Department of Chemistry, University of SargodhaSargodha, Pakistan; ^3^Drug and Herbal Research Centre, Faculty of Pharmacy, Universiti Kebangsaan MalaysiaKuala Lumpur, Malaysia

**Keywords:** *Cydonia oblonga*, phytomedicine, pharmacological attributes, folk medicinal uses, Quince

## Abstract

*Cydonia oblonga* M. is a medicinal plant of family *Rosaceae* which is used to prevent or treat several ailments such as cancer, diabetes, hepatitis, ulcer, respiratory, and urinary infections, etc. *Cydonia oblonga* commonly known as Quince is rich in useful secondary metabolites such as phenolics, steroids, flavonoids, terpenoids, tannins, sugars, organic acids, and glycosides. A wide range of pharmacological activities like antioxidant, antibacterial, antifungal, anti-inflammatory, hepatoprotective, cardiovascular, antidepressant, antidiarrheal, hypolipidemic, diuretic, and hypoglycemic have been ascribed to various parts of *C. oblonga*. The polysaccharide mucilage, glucuronoxylan extruded from seeds of *C. oblonga* is used in dermal patches to heal wounds. This review focuses on detailed investigations of high-valued phytochemicals as well as pharmacological and phytomedicinal attributes of the plant.

## Introduction

Plants are not only a dietary source for both human beings and animals but also safer phytomedicines. Traditionally, phytomedicines have been used to treat various ailments in Unani-tibb, Chinese, and ayurvedic systems of therapies (Gilani and Rahman, 2005; Krishnaswamy, 2008; Muhammad et al., 2014). This curing potential of plants can be supported by numerous scientific evidences (Gilani, 1998; Lattanzio et al., 2009; Banerjee et al., 2011; Russell and Duthie, 2011; Anwar et al., 2016). In response to new challenges in health care, researchers are focusing plants to isolate active phytochemicals (Lattanzio et al., 2009; Banerjee et al., 2011; Russell and Duthie, 2011; Muhammad et al., 2016). The reliability on phytomedicines for treatment of different disorders is greater in present era than never before. In traditional Indian folk medicines, more than 25,000 plant based drug formulations have been documented (Kusari et al., 2014).

*Cydonia oblonga* (Syn: Quince, Bahee Dana, Strythion, and Safarjal), a plant of family *Rosaceae* (Torkelson, 1995; Marwat et al., 2009; Khoubnasabjafari and Jouyban, 2011) is popular for its medicinal, nutritional, and ornamental uses. Its fruit is used in food industry (Usmanghani et al., 1997) as a source of pectin that protects colonal damage in irritable bowel syndrome (IBD) and peptic ulcer (Hamauzu et al., 2008; Minaiyan et al., 2012). The presence of vitamin C and different minerals such as phosphorus, calcium, potassium, sodium, and nitrogen in quince fruit has also been reported (Rop et al., 2011). Seeds of the plant are traditionally utilized for the treatment of diarrhea, cough, dysentery, sore throat, constipation, and bronchitis (Nadkarni, 1976; Duke et al., 2002; Prajapati et al., 2006).

Quince seeds contain sterols, triterpenes, and tannins as active phytochemicals that account for its anti-diarrheal activity (Kirimer et al., 1997; Ammar et al., 2009; Budriesi et al., 2010). The presence of different phenolics, organic acids, and amino acids has also been described in Quince seeds (Silva et al., 2005). Quince leaf extract has been found effective against diabetes, cancer, and hemolysis (Costa et al., 2009; Aslan et al., 2010; Carvalho et al., 2010; Jouyban et al., 2011). The plant also contains an enzyme, phenol peroxidase which decolorizes carcinogenic aromatic dyes in industrial waste water (Nandi et al., 2009; Arabaci and Usluoglu, 2014). Essential oils, phenolic compounds, organic acids, tetracyclic sesterterpenes, and ionone glycosides are present in different parts of quince (De Tommasi et al., 1996; Lutz-Roder et al., 2002; Oliveira et al., 2007, 2008; Osman et al., 2010; Erdogan et al., 2012).

The wide spread medicinal uses of Quince and its valuable phytochemical makeup have attracted our attention to pile up a comprehensive review on its potential bioactive components, bio-medical, and nutritional applications. So far, no comprehensive review has been compiled to describe pharmacological attributes, folk medicinal uses, and phytochemical constituents of Quince in recent years in order to bridge the knowledge gap among researchers.

## Taxonomy and distribution

Quince (Family: *Rosaceae*) is a small plant or shrub with a height of 5–8 and 4–5 m wide. It is the sole member of genus *Cydonia*. Its fruit has bright yellow coloration, acquiring 7–12 cm length, and 6–9 cm width. Fruit has astringent taste, characteristic aroma, and large numbers of plano-convex seeds arranged in two vertical rows. The plant blossoms in spring having light pink flowers with diameter of 5 cm (Gholgholab, 1961). Leaves are elliptical in shape, 6–11 cm long and have white hairs on the surface. It is native to Iran and Turkey, and is cultivated in India, South Africa, Middle East, and Europe (Yildirim et al., 2001; Evans et al., 2002). On the basis of fruit shape, two varieties of Quince are available (*C. oblonga* sub sp. *Maliformis* and *Polyformis*). The fruits of first one are apple shaped whereas second species is pear shaped. The apple shaped fruits have harder flesh with more astringent taste as compared to pear shaped. From toxicology point of view, Quince is regarded as safe however toxic effects may be produced by its seeds only when they are ingested in large quantity due to presence of nitrile components (Huxley et al., 1999). Its fruit is a source of natural phenolic compounds possessing anti-bacterial, anti-oxidant, and anti-ulcerative potential (Wang et al., 2006; Fattouch et al., 2007; Hamauzu et al., 2008).

## Phytochemistry

### Quince fruit and peel

Quince fruit has been extensively consumed as a dietary source. Its fruit is used for preparing jams and jellies (Usmanghani et al., 1997). It is also regarded as an economical and natural source for phenolic constituents (Silva et al., 2004a; Oliveira et al., 2007). The fruit of Quince contains malic acid (1.2 ± 0.8%), reducing sugars (5.0 ± 1.0%), tannins (0.8 ± 0.02%), vitamin C (16.8 ± 0.8 mg/100 g), pectin (1.8 ± 0.1%), and minerals like potassium (248 ± 0.02 mg/100 g), sodium (8.0 ± 0.03 mg/100 g) calcium (18.0 ± 0.02 mg/100 g), and phosphorus (26.0 ± 0.04 mg/100 g; Rop et al., 2011; Sharma et al., 2011). The importance of fruits and vegetables in reducing risks of heart disease, aging, and cancer is well-known (Fattouch et al., 2007). These health benefits are attributed to strong antioxidant potential of phenolic acids and flavonoids present in plants (Silva et al., 2004b).

**Figure 1:**
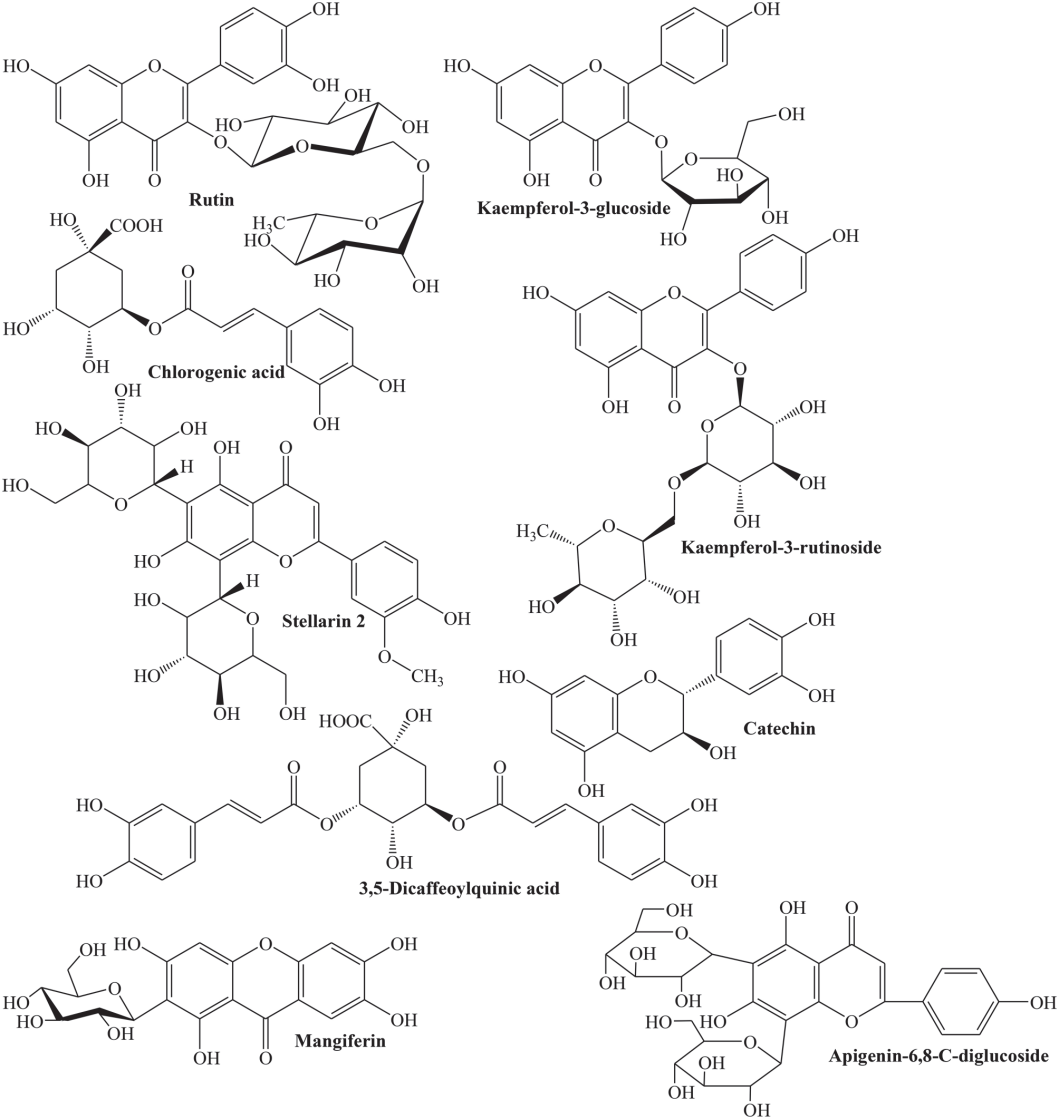
**Some selected polyphenolics isolated from various parts of Quince**.

Considering the medicinal importance of antioxidants, different studies have been carried out to determine phenolic profile and antioxidant potential of Quince fruit. Silva et al. (2002a) revealed that fruit contains famous anti-oxidants such as caffeoylquinic acids (79.6 mg/kg) and rutin (5.5 mg/kg), while its peel is also rich source of caffeoylquinic acid (291.6 mg/kg) along with other important flavonoids such as kaempferol 3-glucoside (92.9 mg/g), quercetin 3-galactoside (100.8 mg/g), and kaempferol-3-rutinoside (61.1 mg/g) (Figure 1). The presence of ascorbic, citric, malic, D-(-)-quinic, fumaric, and L-shikimic acids was also confirmed in both peel and pulp (Silva et al., 2002a).

**Figure 2:**
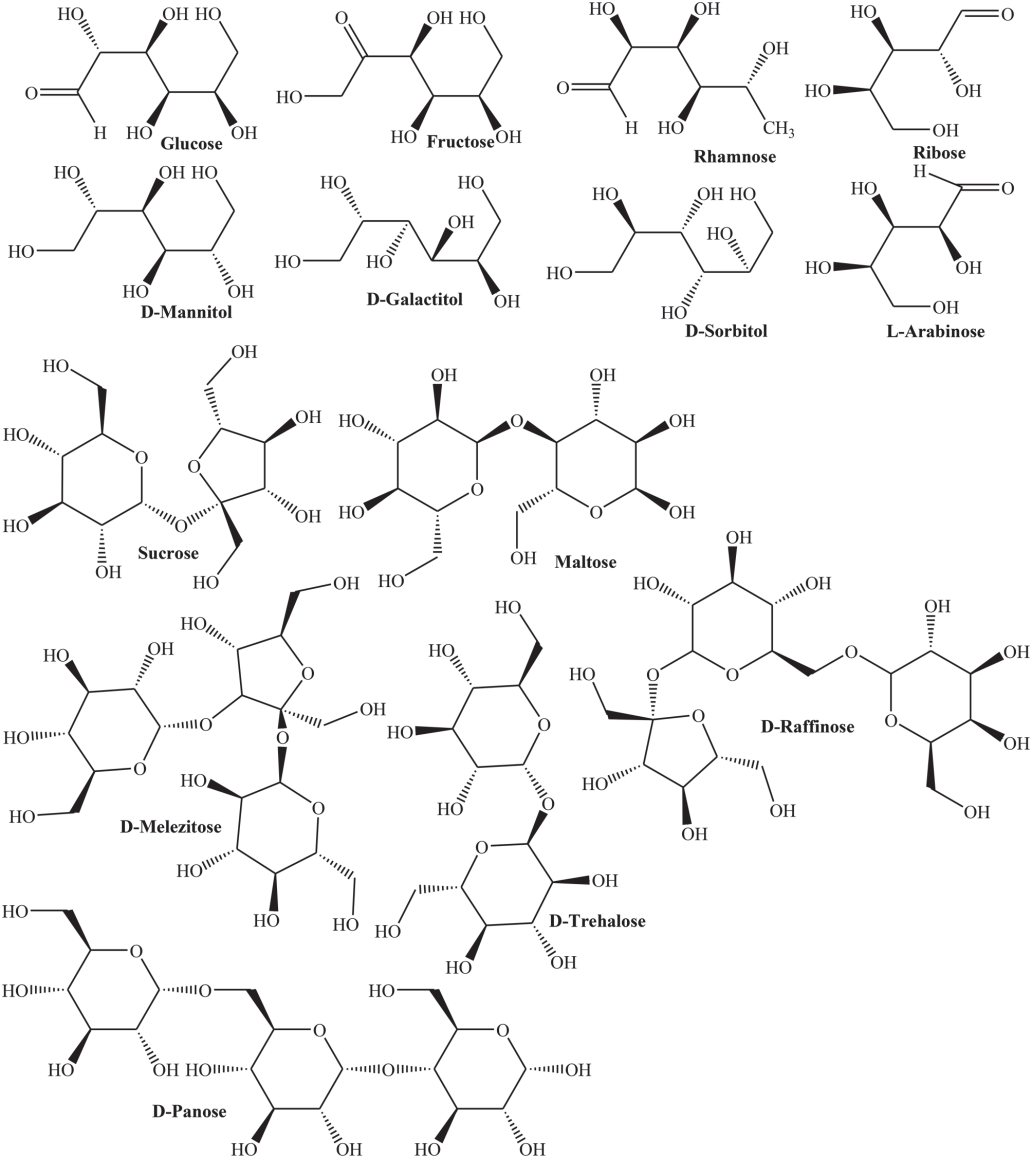
**The structures of important sugars and sugar alcohols from Quince**.

In another study, HPLC revealed rhamnose, mannose, D-glucose, L-arabinose, and galactose monosaccharides in fruit (Hopur et al., 2011). Furthermore, polysaccharides of Quince fruit inhibited activity of tyrosine phosphatase (IC_50_ = 2.07 μg/mL) indicating its capability to treat type 2 diabetes and obesity (Yildirim et al., 2001). Moreover, nectar of three Quince cultivars (Vranjska, Triumph, and Leskovacka) was investigated for contents of D-glucose, fructose, sucrose, maltose, rhamnose, isomaltose, L-arabinose, ribose, D-melezitose, D-panose, D-raffinose, D-trehalose, and sugar alcohols like D-sorbitol, D-mannitol, and D-galactitol using high performance anion exchange chromatography (HPAEC). The results showed that frost in late spring season affected carbohydrate metabolism thus enhancing concentrations of D-trehalose, fructose, ribose, rhamnose, D-raffinose, D-galactitol, and D-mannitol (Figure 2). The study inferred stress induced changes in carbohydrate contents as a mechanism to cope with frost stress (Aksic et al., 2015). The phenolic contents of oven and sun dried peels of Quince were 36.72 ± 3.57, 10.39 ± 1.04 mg/g whereas phenolic contents of oven and sun dried pulps were found in the range of 7.16 ± 0.84 and 19.45 ± 1.26 mg/g, respectively. The radical scavenging activity of sample (0.5 mg) of oven and sun dried fruits (87, 23.20%) and peels (83.35, 28.91%), respectively, clearly suggested that nutritive values of peel and fruit are better preserved in oven drying (Gheisari and Abhari, 2014).

Fattouch et al. (2007) concluded that total phenolic contents (TPC) range from 105 to 157 and 37 to 47 mg/g in peel and pulp, respectively. The chlorogenic acid (5-*O*-caffeoylquinic acid, 37%) and rutin (36%) were major phenolics (Fattouch et al., 2007). In a similar study, Magalhaes et al. (2009) evaluated phenolic profile of fruit (peel, pulp, and seeds) and showed that 5-*O*-caffeoylquinic acid is major phenolic while seeds were rich in 6,8-*di-C*-glucosyl chrysoeriol (stellarin-2). TPC were found to be 6.3, 2.5, and 0.4 g/kg for peel, pulp, and seeds, respectively. This study supported the fact that Quince fruit is mostly consumed in processed form, i.e., jams and jellies rather than fresh form (Magalhaes et al., 2009). Silva et al. (2004b) studied the phenolic, organic, and amino acids contents of Quince before and after jam processing. They also compared composition of peeled and unpeeled fruit jams. The pulp contains 3-, 4-, and 5-O-caffeoylquinic acids, quercitin 3-galactoside, rutin, and 3,5-dicaffeoylquinic acid while in peel kaempferol 3-glucoside, kaempferol 3-rutinoside, kaempferol glycoside, quercitin glycosides acetylated with *p*-coumaric acid, and two acetylated glycosides of kaempferol were identified (Silva et al., 2004b).

**Figure 3:**
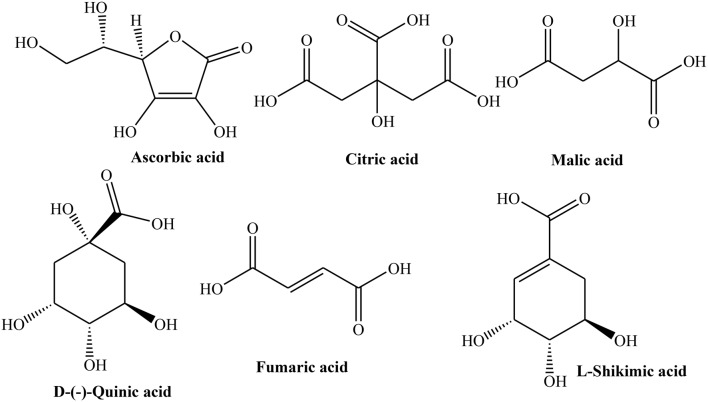
**Structures of some selected acids isolated from various parts of Quince**.

The peeled and unpeeled fruit jams were also analyzed for their phenolic contents. The study inferred that unpeeled jam contained greater flavonoids (19%) than peeled (3%). After processing, concentrations of total phenolics of jam were found 57% instead of actual amount of pulp (50%) used. This might be due to evaporation during thermal processing. Change in 5-*O*-caffeoylquinic acid was also noticed which may be due to isomerization induced by thermal processing. Both pulp and peel contained citric, ascorbic, malic, L-shikimic, and fumaric acids (Figure 3). Amino acid profile of Quince peel and pulp was also similar containing 21 amino acids. L-Aspartic acid, L-glutamic acid, L-cysteine, hydroxyproline, and L-serine constitute 75–85% of total amino acids. The processing resulted in decrease in concentrations of L-tryptophan, L-histidine, and L-glutamic acid due to their thermal degradation (Silva et al., 2004b,c).

Szychowski et al. (2014) showed that both lipophilic and hydrophilic extracts of Quince peel have higher phenolic contents with greater antioxidant activity. Ratio of TPC of Quince peel to pulp (TPC peel/TPC pulp) was about 4.7. Whereas, linoleic acid (54.7%) and oleic acid (35.5%) were predominant fatty acids (Szychowski et al., 2014). Magalhaes et al. (2009) evaluated phenolic contents of Quince fruit peel, pulp, and seeds, and revealed 5-*O*-caffeoylquinic acid as major phenolic acid of peel (57%) and pulp (29%). However, seeds were rich in stellarin-2 (18%). The study concluded that Quince seeds possess relatively lower phenolic contents (0.4 g/kg) of seeds extract whereas peel and pulp have 6.3 and 2.5 g/kg phenolics (Magalhaes et al., 2009). The aqueous methanolic extract of Quince fruit was investigated to contain D-(-)-quinic acid, 5-*O*-caffeoylquinic acid, D-(-)-quinic acid derivatives, proanthocyanin B1, and methyl 5-*O*-caffeoylquinate (Karar et al., 2013). The results of LC-MS analysis showed the presence of 34 polyphenols in Quince fruit aqueous methanolic extract. The phenolic profile included caffeoylquinic acid derivatives, coumaroylquinic acid derivatives, 3-*O*-caffeoyl L-shikimic acid, kaempferol 3-*O*-rhamnoside-7-*O*-glucoside, kaempferol 3-*O*-rutinoside, catechin and catechin derivatives, epicatechin and its derivatives, kaempferol 7-*O*-glucoside, quercetin-3-*O*-glucosyl-7-*O*-rhamnoside, quercetin-3-*O*-glucoside, D-(-)-quinic acid derivatives, apigenin-6,8-*C*-diglucoside, rutin, and mangiferin (Figure 1) (Karar et al., 2013).

Similarly, Quince fruit was observed to contain 26 polyphenolic components including nine flavans-3-ols [procyanidin B2, three procyanidin dimers, one tetramer and three trimmers, and (-)epicatechin], eight hydroxycinnamate derivatives of D-(-)-quinic acid, and nine keampferol and quercetin derivatives. These phenolics and flavan-3-ols constitute 78–94% of total phenolics (Wojdylo et al., 2013). Evaluation of volatile components of whole fruit showed the presence of esters, acetates, and sesquiterpenes. The concentrations of acetates increased with ripeness except (*Z*)-3-hexenyl acetate. Amounts of sesquiterpenes like α-bergamotene and α-farnesene decreased with ripeness. The α-farnesene was the most abundant volatile compound constituting more than 80 and 70% of volatiles of fruit in October and November, respectively. More than 20% of volatile esters like ethyl decanoate (0.2–0.3%), ethyl-2-octenoate (0.2–0.4%), ethyl acetate (0.6–0.8%), and 5-hexenyl acetate (0.6–0.8%) were detected in November but not in October (Tateo and Bononi, 2010). Tsunevay et al. (1983) reported 120 volatile components including alcohols, esters, ketones, aldehydes, and hydrocarbons.

Umano et al. (1986)identified 34 volatile components in Quince peel extract mainly esters that imparts characteristic odor to the fruit. In another study, it was revealed that Quince fruit oil contained 63 volatile components among which 11 aldehydes, 11 ketones, 11 alcohols, 13 esters, and 2 hydrocarbons were identified. Two new compounds *trans*- and *cis*-3-methyl-5-[(E)-3′-methyl-13′-butadien-1′-yl]tetrahydrofuran (*cis* and *trans* marmelo oxide) were characterized using NMR (Tsunevay et al., 1983). Winterhalter and Schreier (1988) revealed the presence of *C*_13_-non-isoprenoids in polar fractions of fruit which includes 3-hydroxy-β-ionol, 4-hydroxy-β-ionol, 4-oxo-β-ionol, 3-hydroxy-β-ionone, 4-hydroxy-β-ionone, 5,6-di-hydroxy-β-ionone, and dehydrovomifoliol. Enzymatic hydrolysis of glycosidic extract of Quince fruit juice and its adsorption studies on Amberlite XAD-2 showed the presence of certain aglycon moieties such as 3-hydroxy-7,8-dihydro-β-ionol, vomifoliol, 3-oxo-α-ionol, and 7,8-di-hydrovomifoliol.

Quince fruit oil was observed to have four bicyclic [4.3.0] nonanes such as 2,2,6,7-tetramethylbicyclo[4,3,0]nona-4,7,9(1)triene, (+) 2,2,6,7-tetramethybicyclo[4,3,0]nona-4,9(1)-dien-8-one, (-) 2,2,6,7-tetramethyl-bicyclo[4,3,0]nona-4,9(1)-dien-7,8-diol, (-) 2,2,6,7 tetramethybicyclo[4,3,0]nona-4,9(1)-dien-8-ol (Ishihara et al., 1986). Analysis of lipophilic fractions (soluble and insoluble in *n*-hexane and acetone) obtained from dried Quince fruit was ended with conclusion that insoluble *n*-hexane extract consisted of *n*-alcohols, saturated *n*-aldehydes, and free alkanoic acids, triterpenoic acids (oleanolic, betulinic, and ursolic acids) whereas, *n*-hexane soluble portion contained glycerides of linoleic acid and oleic acid along with free palmitic acids, oleic, and linoleic acids. In acetone insoluble portion, odd numbered unbranched hydrocarbons (C27, C29, C31) were identified (Lorenz et al., 2008). Carotenoid cleavage enzyme from Quince fruit was isolated (Fleischmann et al., 2002). It was also reported that optimum extraction of pectin (1.83 g/100 g) was achieved at 90°C after 90 min (Acikgoz, 2011). Other studies also reported the presence of pectin in Quince fruit (Rop et al., 2011; Minaiyan et al., 2012).

### Quince leaves

The leaves of quince have medicinal applications such as protective effect on spermatogenesis in hypercholesterolemia (Ashrafi et al., 2013), anti-fungal (Hamid et al., 2013), renoprotective potential (Jouyban et al., 2011), anti-atherogenic, and hepatoprotective potential (Khademi et al., 2013), anti-proliferative effect against colon cancer cells (Carvalho et al., 2010), and antioxidant potential (Costa et al., 2009) owing to presence of valuable bioactives.

The leaves of Portuguese Quince were found to contain 5-*O*-caffeoylquinic acid (36.2%), 3,5-*O*-dicaffeoylquinic acid (3.63%), 3-*O*-caffeoylquinic acid (8.93%), 4-*O*-caffeoylquinic acid (0.45%), quercitin-3-*O*-rutinoside (21.1%), and kaempferol-3-*O*-rutinoside (12.5%), quercitin-3-*O*-galactoside (5.56%), kaempferol-3-*O*-glycoside (8.25%), and kaempferol-3-*O*-glucoside (3.42%) as studied using HPLC/DAD and HPLC/UV (Oliveira et al., 2007). Similarly, various flavonoids such as quercetin-3-*O*-galactoside, quercetin-3-*O*-rutinoside (rutin), kaempferol-3-*O*-glycoside, kaempferol-3-*O*-rutinoside and kaempferol-3-*O*-glucoside, and phenolic acid, 4-*O*-caffeoylquinic acid were identified in methanolic extract of Tunisian Quince leaves. Moreover, rutin (36%) was the most abundant flavonoid found in its leaves (Benzarti et al., 2015). In another study, chlorogenic acid was identified as a major phenolic of Quince leaves methanolic extract (Costa et al., 2009).

Like phenolics, organic acids are also important antioxidant metabolites of plants which make them useful in treatment of various diseases (Silva et al., 2005). Quince leaves from central and northern Portugal contained D-(-)-quinic acid (72.2%), oxalic acid (6.1%), malic acid (7.6%), and citric acid (13.6%) with small amounts of fumaric and L-shikimic acids when analyzed using HPLC/UV method. It was also observed that leaves collected in June and August possess relatively higher acid contents than in October (Oliveira et al., 2008).

**Figure 4:**
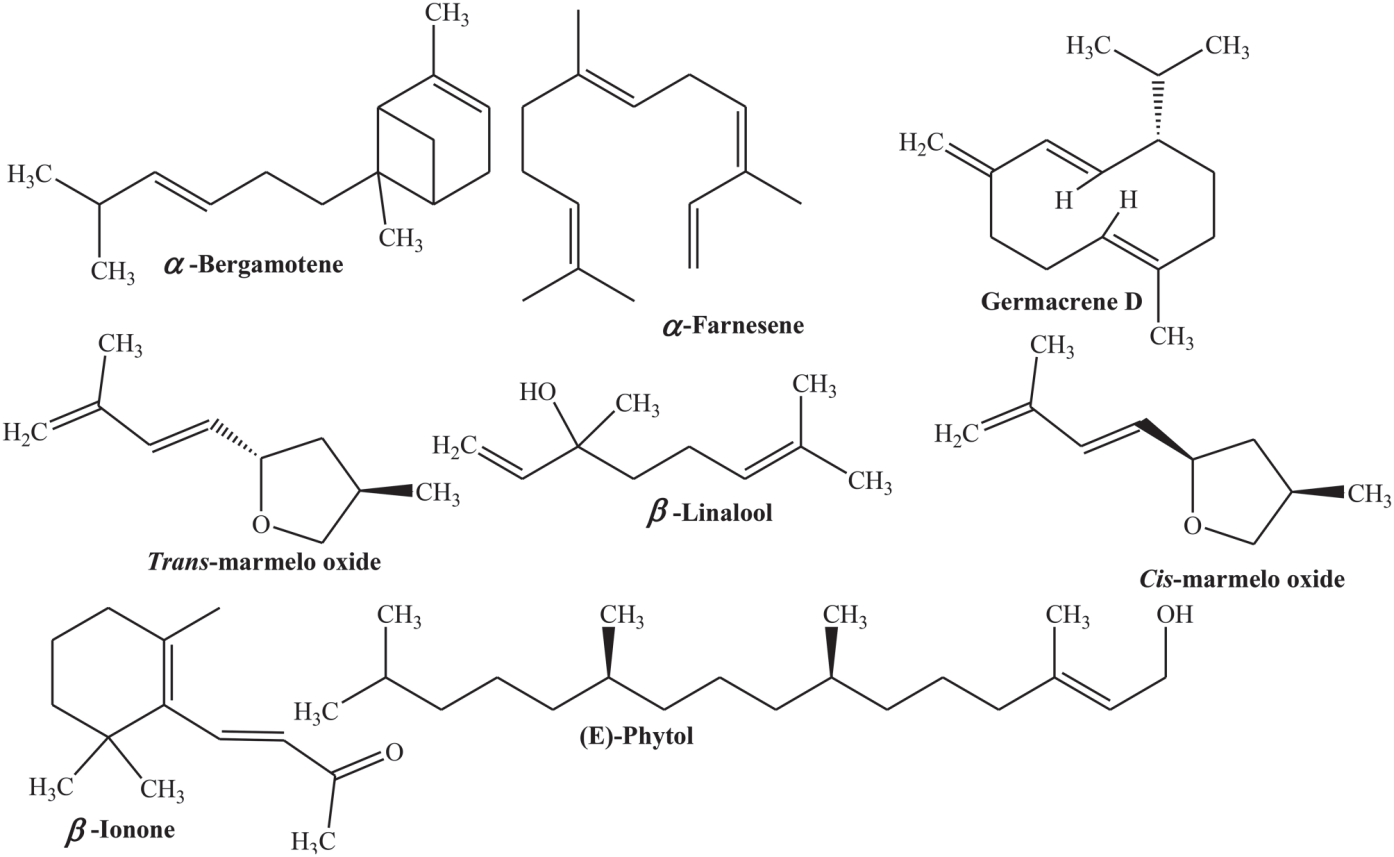
**Structures of selected volatile compounds from Quince**.

Furthermore, GC/MS analysis of aqueous distillate of Quince leaves, collected in flowering (November) and fruiting season (April) was carried out to explore essential oils. The study revealed the presence of 47 and 40 different essential oils which constitutes 95.7 and 64.5% of total oils of leaves gathered during flowering and fruiting seasons, respectively. Aldehydes were 12.8% of total oils in leaves of flowering period followed by fatty acids (hexadecanoic acid 7.2%), monoterpenes (β-linalool 5.7%), and norisoprenoids like (*E*)-β-Ionone (5.1%) and (*E,E*)-α-Farnesene (4.6%). However, leaves of fruiting Quince contained sesquiterpene hydrocarbons (germacrene D 8.6%), benzaldehyde (4.9%), (*Z*)-β-Farnesene (4.8%), (*Z*)-3-Hexenol (3.8%), (E)-phytol (3.1%), (*Z*)-3-hexenal (3.0%) as major constituents of essential oils (Figure 4) (Erdogan et al., 2012).

### Quince seeds

**Figure 5:**
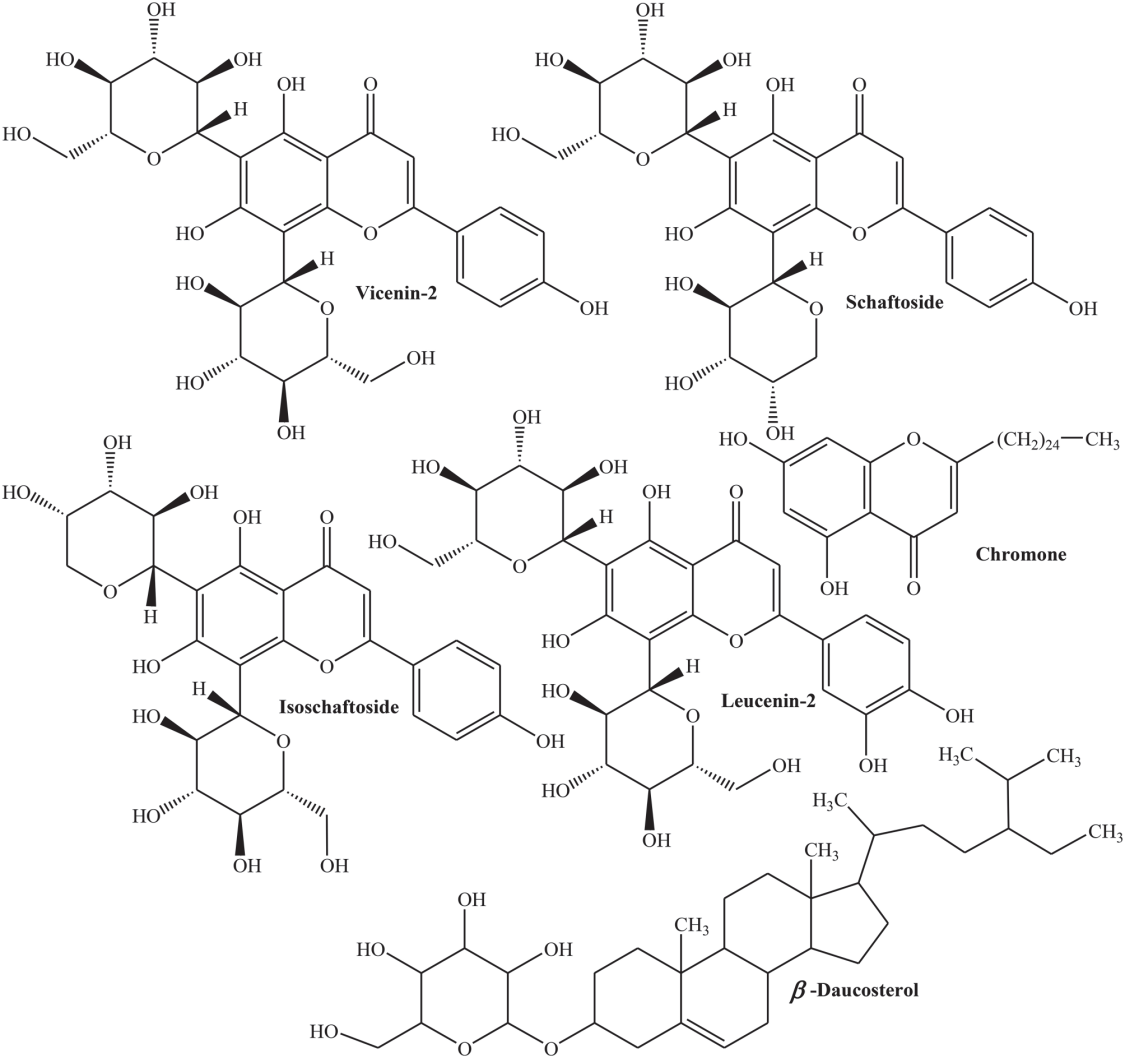
**Selected phytochemicals separated from seeds of Quince**.

Freeze dried seeds of Quince collected from Northern and central Portugal were evaluated for phenolics, free amino acids and organic acids using HPLC/ DAD, GC/FID and HPLC/UV. Different phenolics explored in Quince seeds include 3-,4- and 5*-O-*caffeoyl quinic acids, 3,5-dicaffeoyl quinic acid, apigenin derivatives (vicenin-2, isoschaftoside, and schaftoside), leucenin-2, 6-*C-*pentosyl*-*8-*C-*glucosyl chrysoeriol, 6-*C*-glucosyl-8-*C-*pentosyl chrysoeriol, and stellarin-2 (Figure 5). The flavones are the major part of phytochemical constituents (63–66%) with isoschaftoside (18%), caffeoylquinic acids (35–37%), and 5-*O*-caffeoylquinic acid (19–24%) as prominent flavones. Organic acids identified in Quince seeds are fumaric, L-shikimic, D-(-)-quinic, ascorbic, malic, and citric acids. The total organic acids were found to be 0.8 g/kg of the sample. The amino acids identified in freeze dried seeds are L-glycine, L-valine, L-alanine, L-proline, L-leucine, L-isoleucine, L-glutamic acid, L-serine, L-threonine, L-methionine, L-cysteine, L-phenyl alanine, hydroxyproline, L-asparagine, L-aspartic acid, L-glutamine, ornithine, L-tyrosine, L-histidine, and L-tryptophan which constitute about 1.3–1.7 mg/kg of sample. Moreover, L-aspartic acid, L-glutamic acid, and L-asparagine are about 60–75% of total amino acids (Silva et al., 2005). Phytochemical analysis of ethanolic extract of Quince seeds showed the presence of tannins, glycosides, and phenolic compounds (Al-khazraji, 2013).

Methanolic extract of Quince seeds was evaluated using different chromatographic and spectroscopic techniques and was observed to consist of 6,8-*di-C*-glucosyl luteolin (lucenin-2), 6,8-*di-C*-glucosyl apigenin (vicenin-2), 6,8-*di-C*-glucosyl chrysoeriol (stellarin-2), 6-*C*-arabinosyl-8-*C*-glucosylapigenin (isoschaftoside), 6-*C*-glucosyl-8-*C*-arabinosyl apigenin (schaftoside), 6-*C* pentosyl-8-*C*-glucosyl chrysoeriol, and 6-*C*-glucosyl-8-*C-*pentosyl (Ferreres et al., 2003). A new chromone, 5,7-dihydroxy-2-n-pentacosanylchromen-4-one and three known components such as ursolic acid, tormentic acid, and β-daucosterol have been recently isolated from methanolic extract of Quince seeds (Ghopur et al, 2012).

Quince seed oil was extracted using supercritical fluid (SCF) and ultrasound assisted (UA) extraction techniques to investigate fatty acid profile. The yield of extracted oil was 24.32 and 19.5% with SCF and UA extractions, respectively. The major components were found to be palmitic, linoleic, stearic, oleic and eicosanoic acids (Daneshvand et al., 2012).

Quince seeds extrude mucilage when soaked in water. Earlier investigations carried out to explore different components of mucilage rendered Quince mucilage to be mixture of cellulose and water soluble polysaccharide while acidic hydrolysis revealed the presence of L-arabinose, D-xylose, and aldobiouronic acids (Smith and Montgomery, 1959). In this context, Lindberg et al. (1990) elucidated the structure of water soluble portion of mucilage as partially *O*-acetylated (4-*O*-methyl-D-glucurono)-D-xylan with high proportion of glycuronic acid. On hydrolysis, mucilage yielded arabinose, xylose, mannose, galactose, and glucose. Carbazole method confirmed the existence of uronic acid (35%). Sugar analysis disclosed D-xylose, 4-*O*-methyl glucose, and D-glucose in the mucilage. Methylation analysis unfolded different sugars and their relative proportions in Quince mucilage such as 2,3,4-tri-*O*-methyl-D-xylose, 2,3-di-*O*-methyl-D-xylose, 3-*O*-methyl-D-xylose, and 2,3,4-tri-*O*-methyl-D-xylose whereas branching in (1 → 4)–β-D-xylan backbone at 2-position was observed with 4-O-methyl-α-D-glucopyranosyluronic acid and α-D-glucopyranosyluronic acid (Lindberg et al., 1990).

Study of (4-*O*-methyl-d-glucurono)-D-xylans contents isolated from luffa fruit fibers (*Luffa cylindrical*), jute baste fibers (*Corchorus capsularis*), and Quince seed mucilage depicted variable proportions of D-xylan and 4-*O*-methyl-D-glucoronic acid in jute baste fibers (5:1), luffa fruit (7.5:1), and Quince mucilage (2:1; Vignon and Gey, 1998). They observed that Quince seed mucilage was composed of cellulose microfibrils associated with glucuronoxylans. The presence of 4-*O*-methyl-α-D-glucopyranosyluronic and α-D-glucopyranosyluronic residues (9:1) thus provides additional evidence to earlier findings by Lindberg et al. (1990) Table 1 shows some of the important phytochemicals isolated from various parts of Quince.

**Table 1:** **Selected phytochemicals isolated from various parts of Quince**.

**Plant part used**	**Phytochemicals isolated**
Fruits	3-,4- and 5-*O*-caffeoylquinic acid, 3,5-dicaffeoylquinic acid, quercetin glycoside, kaempferol 3-glucoside, quercetin 3-galactoside, kaempferol 3-rutinoside (Silva et al., 2002a)
	Ascorbic acid, citric acid, malic acid, D-(-)-quinic acid, L-shikimic acid, fumaric acid (Rolandelli et al., 1988)
	Rhamnose, mannose, D-glucose, L-arabinose, and galactose (Hopur et al., 2011)
	Amino acids, i.e., L-aspartic acid, L-glutamic acid, L-cysteine, hydroxyproline, and L-serine (Silva et al., 2004c)
	Fatty acids, i.e., linoleic acid and oleic acid (Szychowski et al., 2014)
	Thirty-four poly phenols were identified including caffeoylquinic acid derivatives, coumaroylquinic acid derivatives (Karar et al., 2013)
	PPO enzyme and phenolic components including flavans-3-ols, including procyanidin B2, procyanidin trimmers, and tetramers, epicatechin, kaempferol, quinic acid, and quercitin derivatives (Wojdylo et al., 2013)
	Volatile components, e.g., actetate like (*Z*)-3-hexenyl acetate, ethyl acetate, 5-hexenyl acetate Volatile esters (ethyl decanoate, ethyl-2-octenoate, sesquiterpenes (α-bergamotene, and α-farnesene), etc., *cis* and *trans* marmelo oxide (Tsunevay et al., 1983; Umano et al., 1986; Tateo and Bononi, 2010)
	C13 non-isoprenoids (Winterhalter and Schreier, 1988)
Leaves	Caffeoyl quinic acid derivatives, quercitin-3-*O*-rutinoside kaempferol-3-*O*-rutinoside quercitin-3-*O*-galactoside, kaempferol-3-*O*-glycoside, kaempferol-3-*O*-glucoside (Oliveira et al., 2007; Benzarti et al., 2015)
	D-(-)-quinic acid, oxalic acid, malic acid, L-shikimic acid, fumaric acid (Oliveira et al., 2008)
	Essential oils, e.g., (*E*)-β-ionone, (*E,E*)-α-farnesene, (*Z*)-3-hexenol (Erdogan et al., 2012)
Seed	Phenolic components, e.g., D-(-)-quinic acid derivatives, apigenin, stellarin, *6-C-*pentosyl*-8-C-*glucosyl chrysoeriol (Silva et al., 2005)
	Tannins, glycosides, and phenolics (Al-khazraji, 2013)
	*C*-glycosylflavons (Ferreres et al., 2003)
	Ursolic acid, tormentic acid, and β-daucosterol and 34 carbon chromone (Ghopur et al, 2012)
	Fatty acids like palmitic acid, linoleic acid, stearic acid, oleic acid, and eicosanoic acid (Daneshvand et al., 2012)
	Sugars like L-arabinose, xylose, mannose, galactose, and D-glucose (Lindberg et al., 1990)

## Folk medicinal uses

Traditionally different parts of plants such as roots, fruit, leaves, and seeds are being used for the treatment of various ailments. The therapeutic potential of different plant parts is attributed to secondary metabolites such as tannins, terpenoids, alkaloids, etc. (Gilani, 1998; Budriesi et al., 2010; Rahmatullah et al., 2010a,b; Ghanbari et al., 2012). Different parts of Quince have been used frequently to get rid of various complications. The seeds are used to treat gastro intestinal (GI) disorders such as constipation, diarrhea, and respiratory tract disorders including cold sores, rhinitis and cough (Nadkarni, 1976; Duke et al., 2002). A prominent formulation, Gencydo^®^ containing aqueous Quince extracts and lemon juice is used for treatment of rhinitis and asthma in Europe. Quince leaf decoction has been traditionally used to cure nervousness, dysuria, insomnia, cough, cold abdominal cramps, diarrhea, fever, and hyperglycemia (Tabata et al., 1993; Tuzlaci and Tolon, 2000; Sezik et al., 2001; Jaladat et al., 2015). The seed mucilage has been used for healing dermal wounds (Ghafourian et al., 2015). Folk medicinal uses of Quince are enlisted in Table 2.

**Table 2:** **Folk medical uses of Quince**.

**Plant part used**	**Uses**
Seeds	Constipation, diarrhea, cold sores, rhinitis, cough, wound healing, dysentery, sore throat (Nadkarni, 1976; Duke et al., 2002; Prajapati et al., 2006; Ghafourian et al., 2015)
Leaves	Nervousness, dysuria, insomnia, cough, cold abdominal cramps, diarrhea, fever, hyperglycemia (Tabata et al., 1993; Tuzlaci and Tolon, 2000; Sezik et al., 2001; Jaladat et al., 2015)
Fruit	Diabetes, urinary complications, respiratory disorders, ulcer, hemolysis (Hamauzu et al., 2008; Carvalho et al., 2010; Jouyban et al., 2011; Minaiyan et al., 2012)

## Pharmacological activities

### Anti-diarrheal activity

Aqueous-methanolic of seeds was studied for its spasmolytic/spasmodic activity in isolated rabbit jejunum and guinea pig ileum. It was observed that seeds extract produced slight prokinetic effect at lower concentrations (0.003–0.03 mg/mL) with EC_50_-value (0.73 mg/mL) and induced muscle relaxation. Moreover, the extract successfully eliminated the KCl induced smooth muscle spasm in rabbit jejunum (EC_50_ 0.86 mg/mL) similar to that of verapamil, a calcium channel blocker. The plant extract also induced atropine sensitive spasmodic effect on isolated ileum of guinea-pig at concentration of 1–10 mg/mL which is about 31.22 ± 3.7% of control, acetyl choline (0.3 μM). This spasmodic effect is attributed to activation of muscarinic receptors, in the gut by the extract like that of acetyl choline. Thus, Quince extract contains spasmodic constituents that relieve constipation. However, plant extract is needed in slightly higher concentration (1–10 mg/mL) for spasmodic effect than spasmolytic action (Janbaz et al., 2013).

### Respiratory and git disorders

Various parts of Quince plant are used to cure respiratory disorders such as asthma, cough, and bronchitis (Nadkarni, 1976; Duke et al., 2002). In this context, seed extract was applied on isolated trachea of rabbit to assess its bronchodilator activity. The results have demonstrated bronchodilator activity of plant extract thus relieving the spasm of tracheal smooth muscles induced by K^+^ and carbachol (CCh). The EC_50_-values were found to be 0.41 and 0.94 mg/mL for K^+^ and CCh, respectively. The results were comparable to verapamil. The bronchodilator activity of plant extract could be attributed to presence of Ca^++^ antagonist components (Janbaz et al., 2013).

Irritable bowel disease (IBD) is a chronic inflammatory disorder that involves inappropriate activation of mucosal immune system in a tenacious manner (McQuaid, 2007; Xavier and Podolsky, 2007). The involvement of ulcerative colitis (UC) and Crohn's disease in IBD is quite possible. The suppression in activities of antioxidant enzymes has also been observed in IBD (Nijveldt et al., 2001). Minaiyan et al. (2012) attempted to treat IBD with hydro-alcoholic extract of Quince fruit (QF) and Quince fruit juice (QFJ) in male Wistar rats at dose of 200, 500, and 800 mg/kg orally, and 200 and 500 mg/kg intra peritoneally for 5 days. Macroscopic and microscopic analysis revealed that both QF and QFJ significantly reduced the colon damage like standard, dexamethasone. It was also showed that QF, QFJ, and dexamethasone were more potent in healing colon damage at 200, 2, and 800 mg/kg oral dose, respectively (Minaiyan et al., 2012). The phenolics like chlorogenic acid and flavonoids such as quercetin, rutin, and kaempferol present in Quince fruit are helpful to repair the colon damage with IBD due to their antioxidant and anti-inflammatory potential (Nijveldt et al., 2001; Silva et al., 2002b; Chagas-Paula et al., 2011; Chauhan et al., 2011; Sato et al., 2011).

Quince fruit also contains pectin in significant amounts which protects colon damage in colitis by triggering colonal cell proliferation (Rolandelli et al., 1988; Roediger, 2010). In a double blind clinical study, the effect of Quince syrup in alleviating gastro-esophageal reflux disease (GERD) symptoms in children (5–18 years old) was assessed. Quince syrup and omeprazole was administered orally to children at dose 0.6 and 1 cc/kg/day in Quince group and omeprazole group, respectively. After 4 and 7 weeks of therapy, age related questionnaires were filled to assess intensity of symptoms, and cumulative symptoms score (CSS) was compared with that of initial base line. A significant reduction of CSS was noticed in Quince group as compared to control group suggesting usefulness of Quince syrup in GERD (Zohalinezhad et al., 2015).

### Anti-bacterial and antifungal activities

*Helicobacter pylori*, a common pathogenic bacterial strain can survive in the acidic environment of stomach and affects 50% population of world (McGowan et al., 1996; Perry et al., 2006). If the infection is prolonged, it may lead to destruction of gastric mucosa and glands, and specialized cells thereby, increasing the risk of gastric cancer (Correa et al., 1975; Slpponen et al., 1985). Common strategy adopted to treat *H. pylori* is a combination therapy using amoxicillin, clarithromycin, and proton pump inhibitors. In case of allergy to penicillin's, amoxicillin is replaced with metronidazole however bismuth compounds are used in case of resistance to aforementioned antibiotics (Fischbach and Evans, 2007; Graham and Shiotani, 2008; Stenstrom et al., 2008). The resistance of microorganisms to antibiotics urges the researchers to discover new phytomedicines from plants which have been used traditionally for curing different ailments (Heep et al., 2000; Nijveldt et al., 2001; Della et al., 2002; Qasim and O'Morain, 2002; Ndip et al., 2007a,b). In one such attempt, Quince juice (10%) inhibited the growth of *H. pylori* (ATCC 43504) on culambia agar media (ZOI 11 mm). A synergistic effect in antibacterial activity of Quince juice was observed with bilberry, black choke berry, red currant juice, green tea, and sweet flag rhizome (Babarikina et al., 2011).

Quince fruit and seeds were extracted with ethanol, acetone and water to study their antimicrobial activity against *Escherichia coli, Klebsiella pneumonia, S. aureus*, and *Enterobacters aerogenes*. Ethanolic extract of Quince seeds was most efficient in inhibiting the growth of bacteria. However, aqueous extracts were found least effective against bacteria (Alizadeh et al., 2013a). Similarly, methanolic extract of Quince seeds inhibited the growth of *S. aureus* (ZOI 12 mm)*, S. epidermidis* (ZOI 15 mm) and *K. pneumonia* (ZOI 8 mm) at concentration of 500, 500, and 250 mg/mL, respectively (Al-khazraji, 2013). Moreover, Quince leaf ethanolic and acetonic extracts reasonable exhibited anti-fungal potential against *Aspergillus niger* (Alizadeh et al., 2013b). Acetone and aqueous extracts of Quince fruit (peel and pulp) depicted antimicrobial activity due to presence of chlorogenic acid (5-*O*-caffeoylquinic acid) along other phenolic components (Fattouch et al., 2007). On similar grounds, Quince seeds ethanolic and acetonic extracts and silver nanoparticles of seeds mucilage have been evaluated for their potential against *S. aureus* that is causative agent for skin burn infections (Hamid et al., 2014). The study was carried out on mice showing better healing of burn wounds infected with *S. aureus* after topical application of ethanolic and acetonic extracts of Quince seeds than that of silver nanoparticles and mupirocin vaseline.

Zsivanovits et al. investigated antibacterial potential of Quince whole fruit variety, *Konstantina polyi* against food born pathogenic bacteria strain (*S. aureus*) using Rapid automated bacterial impedance technique (RABIT; Zsivanovits et al., 2013). The basic principle involved in this technique is to assess the changes in electrolyte composition of nutrient media by growing bacteria (Bolton, 1990). The inhibition in growth of *S. aureus* by Quince fruit extract was assessed comparing integrated area under impedimetric growth curves in different medias i.e. culture media containing only distilled water, culture media containing citric acid and distilled water, and culture media containing Quince fruit extract that were found to be 1,256,014.0 ± 56,474.4, 1,005,672.0 ± 32,851.3, and 8,00,389.5 ± 137.9, respectively, clearly showing anti-bacterial activity of Quince fruit extract (Zsivanovits et al., 2013).

### Cardioprotective and hypolipidemic activities

Cardiovascular diseases (CVD) are associated with diabetes, high blood pressure, atherosclerosis, heart inflammation, and blood clotting. In all aforementioned physiological states, oxidative stress produced by reactive oxygen species (ROS) plays a key role in development of CVD (Griendling and FitzGerald, 2003; Madamanchi et al., 2004; Mueller et al., 2005; Pashkow, 2011). The ROS causing oxidative stress are captured using certain anti-oxidants to prevent CVDs (Rocha et al., 2010). The utilization of vegetables and fruits, the best sources for antioxidants (Murcia et al., 2001) decreased risk of CVDs and other degenerative disorders (Fattouch et al., 2007). It was observed in one study that phenolics particularly 5-*O*-caffeoyl quinic acid present in Quince leaves had immense cardio protective potential as it captured ROS (Vaez et al., 2014). The flavonoids, quercitin, and kaempferol-3-*O*-glucoside (astragalin) and kaempferol-3-*O*-rutinoside in Quince leaves are also cardioprotective (Khoubnasabjafari and Jouyban, 2011). Flavonoids also modulate cardiac inflammation by controlling activation of T cells, B cells, mast cells, neutrophils, and basophils. Thus, Quince leaves could be utilized as natural and economical source that can protect cardiovascular disorders (Middleton and Kandaswami, 1992; Middleton et al., 2000).

WHO defined hypertension as a persistent increase of blood pressure and is one of the major causes of cardiovascular disorders (Rubin et al., 2012). According to WHO report (2002), about 7 million people died of hypertension annually [World Health Organization (WHO), 2002]. Different drug therapies are being used for management of hypertension such as β-blockers, vasodilators, angiotensin converting enzyme inhibitor (ACE) inhibitors, calcium channel blockers, and diuretics with side effects (Ahmad et al., 2005). In a study, it was observed that ethanolic extract of quince leaves and fruit at dose of 80 and 160 mg/kg body weight lowered blood pressure after 4 weeks while captopril (25 mg/kg) after 2 weeks. After 8 weeks, blood pressure was similar in captopril (167 ± 7) and ethanolic extract (166 ± 4) treated rats as compared with model rats (193 ± 7). The effect of aqueous extracts (20, 40, and 80 mg/kg dose) of Quince leaves and fruit increased clotting (1.44, 2.47, and 2.48) and bleeding times (2.17, 2.78, and 3.63) as compared to aspirin (1.91 and 2.58), respectively. The mortality reduction with extracts (27, 40, and 53%) due to pulmonary embolus was promising as compared to aspirin (47%). Thrombolysis was also increased with Quince aqueous extracts (45, 55, and 63%) as compared to aspirin (56%). The results proved the potential use of Quince for prevention of thrombosis and to decrease risks of cardiovascular disorders (Zhou et al., 2014).

Hypercholesterolemia caused by low density lipoproteins (LDL) leads to atherosclerosis which is believed to be the one of the major causes of cardiovascular disorders (Hansson, 2005; Rocha et al., 2010; Moore and Tabas, 2011; Poredos and Jezovnik, 2011). Current treatment strategies employ statins like atorvastatin to decrease the cholesterol levels in the blood with associated risks of muscular toxicity. Therefore, researchers are looking for medicinal plants such as Quince to manage atheroscleroma. Quince has nutritional importance and also regarded as source of phenolics and flavonoids that are medicinally valuable (Oliveira et al., 2007; Costa et al., 2009). Khademi et al. (2013) studied the effect of methanolic fraction of leaf extract on atherosclerosis in white albino rabbits in which atherosclerosis was induced with high cholesterol diet fed for 8 weeks. After 8th week, blood samples were analyzed for serum cholesterol, alanine transaminase (ALT), aspartate transaminase (AST), triglycerides, alanine phosphatase (AP), and histopathology of aorta of normal and high cholesterol fed rabbits. Significant reduction in serum lipids indicates the protective effect of Quince extract on atherosclerosis. Moreover, thickness of atheroma in control and treated group animals was found almost similar, showing usefulness of Quince extract to prevent plaque formation.

Quince fruit aqueous extract was also evaluated for its potential to overcome complications associated with diabetes. The extract at once daily dose of 80, 160 and 240 mg/kg body weight for 6 weeks was orally administered to male Sprague–Dawley rats in which diabetes was induced by single intraperitoneal dose of streptozotocin (60 mg/kg) dissolved in citrate buffer (1 mL, pH 4.5). The results clearly demonstrated that Quince fruit extract successfully reduced total cholesterol level, serum triglycerides, ALT, AST ALP, HDL, LDL, urea, and creatinine (Mirmohammadlu et al., 2015). Quince leaf extract successfully reduced level of total cholesterol (TC), low density lipoproteins (LDL), serum triglycerides (TG), liver stenosis and increased high density lipoproteins (HDL) and lipoprotein lipase (LPL). The extract inhibited the activity of ALT, AST and LPS, whereas activity of superoxide dismutase (SOD), glutathione peroxidase (GSH-PX), and hepatic lipase (HL) was increased in hyperlipidemic rats after 56 days of treatment. The results were comparable with simvastatin except increased LPL and HL quince leaf extract (Abliz et al., 2014).

### Antioxidant and anti-hemolytic activities

Oxidation of substances present in biological membrane due to free radicals is associated with different pathological conditions such as aging and cancer (Halliwell and Gutteridge, 1985, 1986). Human erythrocytes have been extensively used to investigate oxidation process involving biological membrane damage (Girotti et al., 1985; Kobayashi et al., 1985). Oxidation process involves membrane, hemoglobin and over all cellular damage. However, hemoglobin is the main site of damage (Goldberg and Stern, 1977). Free radical scavenging agents such as uric acid, ascorbic acid, chromanol, and vitamin E have been successfully used to reduce erythrocyte hemolysis (Niki et al., 1988). Currently, it has been established that different ailments such as Parkinson's disease, Alzheimer's, cancer, arteriosclerosis, diabetes, and arthritis are associated with generation of free radicals that cause hemolysis (Labat-Robert and Robert, 2014). In a study, phenolics of methanolic extract of Quince fruit seeds, peel, and pulp were isolated using HPLC/UV and evaluated for free radical scavenging abilities using DPPH assay. The potential of extracts to prevent oxidative hemolysis of human erythrocytes induced by 2,2-azobis (2-amidinopropane)-dihydrochloride (AAPH) was also studied. Antioxidant ability of peel (EC_50_ = 0.8 mg/mL) and pulp (EC_50_ = 0.6 mg/mL) extracts was significant and greater than seeds (EC_50_ = 12.2 mg/mL) to prevent erythrocytes hemolysis (Magalhaes et al., 2009). Silva et al. carried out qualitative and quantitative analysis of Quince fruit (pulp and peel) collected from different regions of Portugal and evaluated their antioxidant potential using DPPH assay. It was observed that phenolic fractions possessed strong free radical scavenging activity than that of organic acid fractions and whole methanolic fractions (Silva et al., 2004a). Phenolic composition of fruit and its antioxidant ability provide clear evidence of its medicinal importance. Papp et al. compared phenolic profile and antioxidant potential of Quince peel and fruit of 12 different cultivars and inferred that fruit is a rich source of phenolics with strong antioxidant activity. The cultivars such as “Champion,” “De Husi,” and “Konstantinapolyi” were found best for scavenging free radicals. The analysis of phenolic contents of aqueous alcoholic extracts using Folin–Ciocalteu reagent depicted appreciable amounts of phenolics (8.55 mg GAE/g FW) in fruit (Papp et al., 2013). Similarly, antioxidant potential of Quince fruits pulp and peel of Tunisian Quince predicted that peel has higher radical scavenging activity than pulp (Fattouch et al., 2007).

The quantitative assessment of TPC was found to be 235.66 GAE/g (gallic acid equivalent) and 17.6 QE/g (quercitin equivalent) in methanolic extract of Quince leaves. The antioxidant capacity of Quince leaf extract (IC_50_ = 36.5 μg/mL) was found almost similar to standard with BHT (38.4 μg/ mL; Benzarti et al., 2015). Quince leaves methanolic extract was evaluated for its potential to alleviate hematological changes induced by Ultra violet (UV) radiations in cat fish (*Clarias gariepinus*). It was observed that leaf extract significantly protected RBCs from radiation damage and strengthened immune system raising WBCs and lymphocyte count. Biochemical parameters such as blood glucose, ALT, AST, plasma proteins, and serum creatinine were also significantly lowered by extract (Osman et al., 2010).

A comparative investigation of antioxidant and anti-hemolytic potential of methanolic extract of Quince leaf extract and green tea was carried out (Costa et al., 2009). Phenolic profile of Quince leaf was evaluated using HPLC/UV showing 5-*O-*caffeoylquinic acid as its major component.

Leaf extract of Quince exhibited comparable ability to reduce DPPH free radicals with that of green tea with half maximal effective concentrations (EC_50_ = 12.7 ± 0.1 and 21.6 ± 3.5 μg/ml), respectively, for green tea and methanolic extract of Quince leaves. Both Quince (IC_50_ = 30.7 ± 6.7 μg/ml) and green tea (24.3 ± 9.6 μg/ml) extracts protected erythrocytes from DPPH radicals induced hemolysis (Costa et al., 2009). Antioxidant property of lipophilic extract of Quince fruit and aqueous fermented extract has been reported by Pacifico et al. (2012). Aqueous fermented extract scavenged DPPH free radicals (ID_50_ = 68.8 μg/mL) and successfully prevented the formation of thiobarbituric acid reactive species at very low concentration (73.7 μg/mL). However, lipophilic extract of Quince was more efficient in preventing production of superoxide radicals (ID_50_ 48.9 μg/ML).

### Anti-allergic and anti-inflammatory activities

Allergy is basically hypersensitivity of immune system to harmless substances like dust, pollens, animal dander, medicines, bee sting, etc. They may enter in the body via skin, inhalation, injection, and ingestion leading to activation of immune system. These allergic responses, if not properly managed may produce certain disorders like dermatitis, rhinitis, anaphylactic shock, and asthma. Different drug therapies to treat allergic disorders including antihistamines, immunosuppressant, and corticosteroids are followed by adverse effects like hypertension, diabetes, osteoporosis, and growth retardation (De Benedictis and Bush, 2012). Thus, researchers are trying to explore plants and food materials with anti-allergic potential with no side effect. In this context, anti-allergic effect of preparations from lemon (*Citrus medica* L.) and Quince fruit were investigated. The extracts showed no anti-allergic effect individually. However, the synergistic effect of both the extracts reduced the degranulation of basophils, production of interleukins IL-8 and tumor necrosis factor (TNF-α) from human mast cells significantly (Huber et al., 2012). LC-MS analysis showed the presence of eriocitrin and neochlorogenic acid as major phenolics in citrus and Quince extracts, respectively.

Gencydo^®^, a combination of lemon (*Citrus limon*) juice and Quince fruit extract is used traditionally to treat allergic rhinitis or asthma. In a research, it was observed that Gencydo^®^ caused reduction of histamine, IL-8, and TNF-α release from mast cells induced by Immunoglobulin-E(IgE) and phorbolmyristate acetate (PMA/A23187) in allergic disorders. Furthermore, Gencydo^®^ also blocked eotaxin release from human bronchial epithelial cells. The study supported the use of Gencydo^®^ for the treatment of allergic reactions (Grundemann et al., 2011). Hot water extract of Quince fruit was evaluated for alleviation of type-I allergy (atopic dermatitis) in NC/Nga mice, divided into control and treated groups. Control group was fed with AIN-93M diet and treated groups received 2.5 and 5% hot water Quince fruit extract for 8 weeks. It was found that control group developed skin dermatitis. Moreover, treated group mice have low IgE level especially with 5% hot water Quince fruit extract (994 ± 205 ng/mL) as compared to control (1635 ±289 ng/mL). The study revealed the anti-allergic potential of Quince fruit (Shinomiya et al., 2009). In another study, hot water extract of Quince fruit was found effective against IgE stimulated late phase allergic reactions of mast cells (Kawahara and Lizuka, 2011).

Anti-inflammatory and anti-allergic role of Quince fruit peel phenolics has been investigated after aggravating inflammation in human THP-1 cell line by lipopolysaccharide (LPS). Quince fruit peel extract significantly inhibited the release of inflammatory mediators such as cytokines (TNF-α) and interleukins (IL-8) by inducing release of Interleukin-10 and Interleukin-6 from mast cells. The study also exploited that poly-phenolic extract from Quince peel also inhibited the activation of pro-inflammatory effectors cells by LPS (Essafi-Benkhadir et al., 2012). Anti-inflammatory activity of Quince leaf ethanolic extract was also reported by Ahmed and Bastawy (2014). Ethanolic extract of Quince leaves was administered orally at concentrations of 25, 50, and 100 mg/kg 1 h prior to topical administration of arachidonic acid (2%) to each ear and 0.1 mL of carrageenan injection to sub-planer region of paw for induction of paw edema (Winter et al., 1962). Quince leaf extract effectively alleviated symptoms of carrageenan induced paw edema and arachidonic acid induced ear edema in rats (Ahmed and Bastawy, 2014).

### Spermatogenesis and genoprotective effects

Numerous studies carried out in human beings have explained the effect of hyperlipidemia and hypercholesterolemia on spermatogenesis. It has been verified that raised blood cholesterol and serum lipids results in male infertility (Jones et al., 1979; Padron et al., 1989; Ramirez-Torres et al., 2000). The effect of Quince leaves decoction on protection of testes and restoration of spermatogenesis has been studied in hypercholestrolemic rabbits. Hypercholesterolemia was induced with high cholesterol diet before administering leaf decoction (100 mL) for 12 weeks. The histopathology of testes showed an increase in intertubular connective tissues and thickening of tunica albuginea in all animals of untreated group however histological picture of treated group was comparable with that of control. The mean Johnsen's score of untreated group (4.20 ± 1.92) was also lower than treated (7.33 ± 0.52) and control group (7.05 ± 0.07). The study ended with conclusion that Quince leaves decoction had protective effect on spermatogenesis (Ashrafi et al., 2013).

DNA damage either single or double strand breaks is imposed by numerous factors and is the leading cause of cancer (Brem and Hall, 2005). Breaks in double strand of DNA may lead to serious consequences such as genetic mutations followed by cancer and uncontrolled cellular death (Jackson and Bartek, 2009). Biomaterials such as fruits and vegetables are rich in polyphenolics that possess antioxidant, anti-mutagenic, anti-inflammatory, and anti-carcinogenic potential (Ferguson, 2001). Mobarakeh et al. (2015) evaluated genoprotective potential of aqueous and hydroalcoholic extracts of Quince fruit against genotoxic effect of methylmethanesulfonate (MMS) on human hepatoma cells (HepG2 cells) using comet assay. For this purpose, HepG2 cells were incubated with 100 μM concentration of MMS for 1 h, followed by incubation with Quince extracts (10, 50, 100, and 500 μg/mL) for 2 h. It was revealed that tail length, %DNA contents in tail and tail moment of HepG2 was significantly decreased in both aqueous and hydroalcoholic extracts showing genoprotective effect of Quince fruit.

### Wound healing and anti-proliferative activities

Wound healing is a complex process and could be divided into four distinct phases on physiological grounds which are inflammations, haemostasis, proliferation, and tissue repairing (Clark, 1996). In the whole process of tissue repair, fibroblasts play key role to break clots, in the development of extracellular matrix and collagen along with contraction of wound (Stortelers et al., 2008). Traditionally, Quince seeds mucilage has been used as folk remedy for wound healing (Hemmati and Mohammadian, 2000). The effect of Quince seed mucilage on proliferation of human skin fibroblasts was evaluated to study the mechanism of wound healing. Different concentrations (50, 100, 200, 400 μg/mL) of mucilage were applied to human skin line culture and effect of mucilage was observed after 12, 24, 48, and 72 h. The investigations cleared that Quince mucilage geared up proliferation of human skin fibroblasts after 48 h even at low concentration (50 μg/mL) as studied using microculturetetrazolium assay (Ghafourian et al., 2015). In another study, evaluation of ethanolic extract of Quince seeds for healing second degree burn wounds was carried out on mice showing that 1% ointment of Quince seed extract produced 99.5% of wound healing as compared to sulfadiazine standard (92.97%; Tajoddini et al., 2013). Similarly, Quince seeds methanolic and acetonic extracts, and silver nanoparticles of mucilage were found effective against wounds infected with *S. aureus* (Alizadeh et al., 2013a). The effectiveness of Quince seeds mucilage for skin wound healing has also been justified applying 5, 10, and 20% Quince seeds mucilage cream (QMC) in eucerin base on skin wounds of white Iranian rabbits. QMC (20%) cream healed wounds completely in 13 day treatment (Tamri et al., 2014). Dermal patches of Quince mucilage were prepared and evaluated for mechanical, microstructural, antioxidant, anti-bacterial, physical, and thermal parameters by incorporation of 1, 1.5, and 2% v/v oregano essential oils (Jouki et al., 2014). Fekri et al. (2008) analyzed moisture content, percentage yield, proteins, and ash contents of mucilage. The moisture content, percentage yield, proteins, and ash contents were found to be 4.38, 10.97, 20.9, and 8.24%, respectively.

Anti-proliferative potential of methanolic extract of Quince leaves and its phenolic profile has been evaluated by Carvalho et al. It was observed that Quince leaves extract have concentration dependent anti-cancer activity against human colon cancer cells (IC_50_ = 239.7 ± 43.2 μg/mL). The analysis of phenolic profile using HPLC/DAD showed 5-*O-*caffeoylquinic acid as the major phenolic constituents in Quince leaves. They also explored anti-proliferative potential of methanolic extract of Quince fruit (peel, pulp, and seeds) against human colon and kidney cancer cells. It was observed that fruit and seeds extracts are highly effective against renal cancer cells at concentration of 500 μg/mL. However, anti-proliferative activity against colon cancer cells was not observed (Carvalho et al., 2010). Similarly, lipophilic Quince wax extract and Quince aqueous fermented extract exhibited anti-proliferative potential against Hela cell line, Hep G2, and A549 human cancer cell lines. It was also found that Quince aqueous fermented extract was more potent anti-proliferative agent (Pacifico et al., 2012).

### Anti-diabetic and renal protective activities

Diabetes is a common disease that has affected nearly 10% of population in the world (Bilbis et al., 2002; Irshaid et al., 2012). The disease is characterized by disturbances in fat and protein metabolism leading to hyperlipidemia (Ashraf et al., 2013). Diabetes is also associated with vascular complications which are the major cause of mortality in diabetic patients (Campos, 2012). Quince leaves were extracted with methanol to evaluate its anti-diabetic potential. The findings of study showed that oral administration of extract at dose of 500 mg/kg body weight decreased blood glucose level (33.8%) considerably in streptozotocin induced diabetic rats after 5 days (Aslan et al., 2010).

Hypercholesterolemia not only causes renal injury (Mune et al., 1999; Attia et al., 2002) but also leads to proteinuria, glumerulosclerosis, and masangial cell damage (Eddy, 1996; Joles et al., 2000). This malfunction of glomerulus can be eliminated using antioxidants (Kasiske et al., 1988). Considering, the presence of phenolics in Quince fruit and leaves, and its traditional use to treat different ailments (Fattouch et al., 2007; Oliveira et al., 2007), renoprotective potential of Quince leaves decoction has been evaluated in white New Zealand male rabbits divided into three groups (Jouyban et al., 2011). Before feeding with normal diet for 6 weeks, group I was fed with high cholesterol diet and group II was administered high cholesterol diet with Quince leaf decoction for 6 weeks whereas group III was treated as control group. After this diet, all animals were shifted to their normal diet for another 6 weeks. At the end of this study (12 weeks), urine samples from all groups were collected and ratio of urine protein to creatinine was calculated. All animals were sacrificed to assess the kidney damage due to high cholesterol diet and observed that Quince leaf decoction has significantly prevented renal injury in hypercholesterolemic rabbits that might be due to anti-oxidant activity of phenolics present in Quince (Jouyban et al., 2011). The renal protective effect of antioxidants and lipid lowering agents in hypercholesterolemia has been well-established (Kasiske et al., 1988; Trovato et al., 2010). Pharmacological activities of Quince are shown in Table 3.

**Table 3:** **Summary of pharmacological attributes of Quince**.

**Pharmacological activities**	**Plant part used**	**Phytochemicals**
Anti-diarrheal	Seeds aqueous methanolic extract (Janbaz et al., 2013)	–
Anti-asthma activity	Seeds extract (Janbaz et al., 2013)	–
Protective effect on spermatogenesis	Quince leaf decoction (Ashrafi et al., 2013)	–
Decolorization of dyes	PPO enzyme from Quince leaves (Arabaci and Usluoglu, 2014)	–
Renal protection	Leaves decoction (Jouyban et al., 2011)	–
Anti-bacterial activity	Quince fruit (Babarikina et al., 2011)	Chlorogenic acid (5-*O*-caffeoylquinic acid; (Fattouch et al., 2007))
	Quince seed and fruit extracts (Alizadeh et al., 2013a)	
Anti-fungal	Quince seeds extract (Alizadeh et al., 2013b)	–
	Quince leaf extracts (Hamid et al., 2014)	
	Quince whole fruit (Zsivanovits et al., 2013)	
	Quince fruit (peel and pulp; (Fattouch et al., 2007))	
Anti-proliferative effect	Quince fruit (peel, pulp and seeds, and leaves; (Carvalho et al., 2010))	5-*O-*caffeoylquinic acid (Carvalho et al., 2010)
Prevention of atherosclerosis	Leaf extract (Khademi et al., 2013; Vaez et al., 2014)	–
Cardiovascular protection	Leaf extract (Khademi et al., 2013; Vaez et al., 2014)	5-*O*-caffeoyl quinic acid, flavonoids, Quercetin and kaempferol-3-*O*-glucoside (astragalin) and kaempferol-3-*O*-rutinoside (Khoubnasabjafari and Jouyban, 2011; Vaez et al., 2014)
Activity against IBD	Quince fruit (Minaiyan et al., 2012)	Quercetin, rutin, kaempferol ((Nijveldt et al., 2001; Silva et al., 2002b; Chagas-Paula et al., 2011; Chauhan et al., 2011; Sato et al., 2011);)
GERD	Quince syrup (Zohalinezhad et al., 2015)	–
Wound healing	Seeds mucilage (Ghafourian et al., 2015)	–
	Seeds ethanolic extract (Tajoddini et al., 2013)	
Anti-hemolytic effect	Peel and pulp (Magalhaes et al., 2009)	Uric acid, ascorbic acid, chromanol and vitamin E (Niki et al., 1988), 5-*O-*caffeoylquinic acid (Costa et al., 2009)
	Quince leaf methanolic extract (Pacifico et al., 2012)	
Antioxidant effect	Quince leaf methanolic extract (Pacifico et al., 2012)	Quercetin, rutin, kaempferol (Nijveldt et al., 2001; Silva et al., 2002b), caffeoylquinic acids (Silva et al., 2002a)
	Fruit (peel, pulp, and seeds; (Silva et al., 2004a; Szychowski et al., 2014))	
Anti-allergic effect	Quince fruit extract (Huber et al., 2012)	–
	Hot water fruit extract (Shinomiya et al., 2009)	
Anti-inflammatory	Quince peel (Essafi-Benkhadir et al., 2012)	Quercetin, rutin, kaempferol (Nijveldt et al., 2001; Silva et al., 2002b)
	Quince leaf ethanolic extract (Ahmed and Bastawy, 2014)	
Anti-diabetic	Leaf methanolic extract (Aslan et al., 2010)	–
	Quince fruit (Mirmohammadlu et al., 2015)	
Hepatoprotective	Quince leaf (Abliz et al., 2014)	–
Anti-hypertensive	Quince leaf methanolic extract (Zhou et al., 2014)	–
Anti-thrombotic	Quince leaf and fruit aqueous extracts (Zhou et al., 2014)	–
Geno-protectiuve	Aqueous and hydro-alcohlic extracts of Quince fruit (Mobarakeh et al., 2015)	–
UV protective effect	Quince leaf methanolic extract (Osman et al., 2010)	–

## Treatment of industrial wastewater

The utilization of various synthetic dyes in food, leather, pharmaceutical, rubber and cosmetic industries have been increased for the last many years (Patel and Patel, 2012) thus exposing living organisms to various diseases like cancer (Nandi et al., 2009). Nowadays, researchers are interested to get rid of these industrial wastes using inexpensive and energy efficient enzymatic degradation method than chemical methods (Eichlerova et al., 2006; Pengthamkeerati et al., 2010; Hamid et al., 2013). Oxido-reductase enzymes such as polyphenoloxidases (PPO) and peroxidases of microbial and plant origin are being widely used (Husain and Jan, 2000; Bhunia et al., 2001). In a study, the comparison of free and immobilized PPO on calcium alginate beads obtained from Quince leaves was carried out. The stability of free and immobilized enzyme was evaluated at different pH range (3.5–9.0). The optimum stability was observed at pH 7.5 however immobilized enzyme was found to be more stable at different pH ranges than free enzyme. The immobilized PPO was found to be more thermally stable than free. The decolorization activity of both free and immobilized PPO was optimum between pH (4.0–7.0) after which it was noticed to drop off (Arabaci and Usluoglu, 2014).

## Conclusions and future prospects

*Cydonia oblonga* is medicinal plant of family *Rosaceae* which has attracted the researchers owing to its folk medicinal uses and high-valued bioactives. Besides pharmaceutical attributes, the plant is also popular because of renoprotecticve, hepatoprotective, antidiabetic, anti-proliferative, anti-hemolytic, anti-inflammatory, anti-allergic, geno-protective, and cardioprotective activities. Protective effect of its leaves on male fertility has been established. Hence, there is immense need to isolate potential bioactives from Quince for the development of new safer and economical drugs.

Moreover, plant is rich source of pectin used in food industry in the preparation of jams and jellies. The plant also contains PPO enzyme which is used to decolorize industrial waste thus providing cost effective alternate to treat industrial water. The plant should be cultivated to isolate pectin and PPO enzyme on commercial basis. Glucuronoxylan polysaccharide from seeds of plant has been used for preparation of dermal films to cure wounds. Cytotoxic investigations are required to establish its safety profile to prepare dermal patches. Furthermore, glucuronoxylan could be the potential candidate for controlled/sustained/targeted drug deliveries after toxicological studies.

Resistance of microorganisms to antibiotics is an emerging issue. Thus, there is a need to develop new antibiotics for the treatment of various ailments. Various literature reports indicated that Quince is rich in microbistatic agents and can be the choice for the isolation of new phytochemicals to introduce new drugs. Several cytotoxic studies revealed that plant has a potential to cure cancer. However, further *in vivo* models should be employed to confirm its anti-proliferative tendency before human trials. The isolation of active anti-cancerous secondary metabolites is also demanding. As a functional food, Quince is rich in minerals like sodium, potassium, phosphorous, etc. and essential oils. So there is a need to standardize and validate its medicinal applications as potential source for nutraceuticals.

## Author contributions

All authors listed, have made substantial, direct and intellectual contribution to the work, and approved it for publication.

### Conflict of interest statement

The authors declare that the research was conducted in the absence of any commercial or financial relationships that could be construed as a potential conflict of interest.

## Figures and Tables

**Figure:**
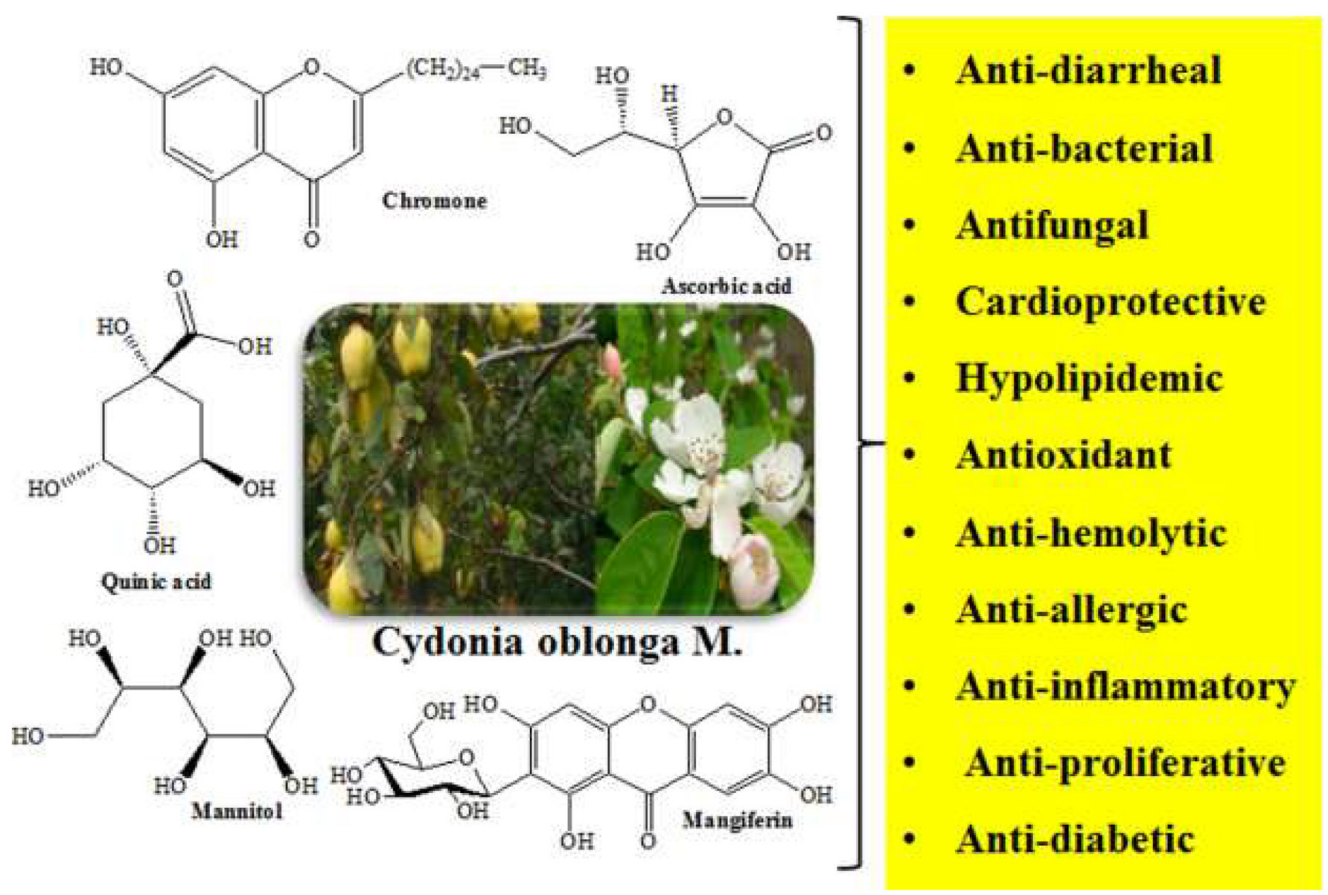


## References

[B1] Abliz A. Aji Q. Abdusalam E. Sun X. Abdurahman A. Zhou W. (2014). Effect of *Cydonia oblonga* Mill. leaf extract on serum lipids and liver function in a rat model of hyperlipidaemia. J. Ethnopharmacol. 151, 2970–2997. 10.1016/j.jep.2013.12.010 24342780

[B2] Acikgoz C. (2011). Extraction and characterization of pectin obtained from Quince fruits (*Cydonia vulgaris pers*) grown in Turkey. Asian J. Chem. 23, 149–152.

[B3] Ahmad M. Aria J. Mosadegh J. (2005). Noscapine suppresses angiotensin converting enzyme inhibitors-induced cough. Nephrology 10, 348–350. 10.1111/j.1440-1797.2005.00429.x 16109080

[B4] Ahmed M. M. Bastawy S. (2014). Evaluation of anti-inflammatory properties and possible mechanism of action of Egyptian quince (*Cydonia oblonga*) leaf. Egypt. J. Biochem. Mol. Biol. 32, 190–205.

[B5] Aksic M. F. Tosti T. Nedic N. Markovic M. Licina V. Milojkovic-Opsenica D. (2015). Influence of frost damage on the sugars and sugar alcohol composition in quince (*Cydonia oblonga* Mill) floral nectar. Acta Physiol. Plant. 37, 1–11. 10.1007/s11738-014-1701-y

[B6] Alizadeh H. Ajalli M. Hossein H. (2013b). Antifungal effect of Cydonia oblonga extracts on Aspergillus *niger*. Jundishapur J. Microbiol. 2013, p4.

[B7] Alizadeh H. Rahnema M. Semnanai S. N. Hajizadeh N. (2013a). Detection of compounds and antibacterial effect of Quince (*Cydonia oblonga* Miller) extracts *in vitro* and in vivo. J. Biol. Active Prod. Nat. 3, 303–309. 10.1080/22311866.2013.817731

[B8] Al-khazraji S. K. (2013). Phytochemical screening and antibacterial activity of the crude extract of *Cydonia oblonga* seeds. Glob. Adv. Res. J. Microbiol. 2, 137–140.

[B9] Ammar S. Edziri H. Mahjoub M. A. Chatter R. Bouraoui A. Mighri Z. (2009). Spasmolytic and anti-inflammatory effects of constituents from Hertia cheirifolia. Phytomedicine 16, 1156–1161. 10.1016/j.phymed.2009.03.012 19403291

[B10] Anwar F. Muhammad G. Hussain M. A. Zengin G. Alkharfy K. M. Ashraf M. (2016). *Capparis spinosa* L.: a plant with high potential for development of functional foods and nutraceuticals/pharmaceuticals. Int. J. Pharmacol. 12, 201–219. 10.3923/ijp.2016.201.219

[B11] Arabaci G. Usluoglu A. (2014). The enzymatic decolorization of textile dyes by the immobilized polyphenol oxidase from Quince leaves. Sci. World J. 2014:685975. 10.1155/2014/685975 24587743 PMC3918733

[B12] Ashraf H. Heidari R. Nejati V. Ilkhanipoor M. (2013). Effects of aqueous extract of *Berberis integerrima* root on some physiological parameters in streptozotocin-induced diabetic rats. Iran. J. Pharm. Res. 12, 425–434. 24250618 PMC3813236

[B13] Ashrafi H. Ghabili K. Alihemmati A. Jouyban A. Shoja M. M. Aslanabadi S.. (2013). The effect of quince leaf (*Cydonia oblonga* Miller) decoction on testes in hypercholesterolemic rabbits: a pilot study. Afr. J. Tradit. Complement. Alter. Med. 10, 277–282. 10.4314/ajtcam.v10i2.12 24146451 PMC3746575

[B14] Aslan M. Orhan N. Orhan D. D. Ergun F. (2010). Hypoglycemic activity and antioxidant potential of some medicinal plants traditionally used in Turkey for diabetes. J. Ethnopharmacol. 128, 384–389. 10.1016/j.jep.2010.01.040 20100559

[B15] Attia D. M. Ni Z. N. Boer P. Attia M. A. Goldschmeding R. Koomans H. A.. (2002). Proteinuria is preceded by decreased nitric oxide synthesis and prevented by a NO donor in cholesterol-fed rats. Kidney Int. 61, 1776–1787. 10.1046/j.1523-1755.2002.00313.x 11967027

[B16] Babarikina A. Nikolajeva V. Babarykin D. (2011). Anti-*Helicobacter* activity of certain food plant extracts and juices and their composition in vitro. Food Nut. Sci. 2, 868–877. 10.4236/fns.2011.28118

[B17] Banerjee G. Car S. Scott-Craig J. S. Hodge D. B. Walton J. D. (2011). Alkaline peroxide pretreatment of corn stover: effects of biomass, peroxide, and enzyme loading and composition on yields of glucose and xylose. Biotech. Biofuels. 4, 16–30. 10.1186/1754-6834-4-16 21658263 PMC3123552

[B18] Benzarti S. Hamdi H. Lahmayer I. Toumi W. Kerkeni A. Belkadhi K. (2015). Total phenolic compounds and antioxidant potential of quince (*Cydonia oblonga* Miller) leaf methanol extract. Int. J. Inov. Appl. Stud. 13, 518–526.

[B19] Bhunia A. Durani S. Wangikar P. P. (2001). Horseradish peroxidase catalyzed degradation of industrially important dyes. Biotech. Bioeng. 72, 562–567. 10.1002/1097-0290(20010305)72:5<562::AID-BIT1020>3.0.CO;2-S 11460246

[B20] Bilbis L. S. Shehu R. A. Abubakar M. G. (2002). Hypoglycemic and hypolipidemic effects of aqueous extract of *Arachis hypogaea* in normal and alloxan-induced diabetic rats. Phytomedicine 9, 553–555. 10.1078/09447110260573191 12403165

[B21] Bolton F. J. (1990). An investigation of indirect conductimetry for detection of some food-borne bacteria. J. Appl. Bacteriol. 69, 665–661. 10.1111/j.1365-2672.1990.tb01559.x 2126006

[B22] Brem R. Hall J. (2005). XRCC1 is required for DNA single-strand break repair in human cells. Nucleic Acids Res. 33, 2512–2520. 10.1093/nar/gki543 15867196 PMC1088068

[B23] Budriesi R. Ioan P. Micucci M. Micucci E. Limongelli V. Chiarini A. (2010). Stop Fitan: antispasmodic effect of natural extract of chestnut wood in guinea pig ileum and proximal colon smooth muscle. J. Med. Food 13, 1104–1110. 10.1089/jmf.2009.0210 20626243

[B24] Campos C. (2012). Chronic hyperglycemia and glucose toxicity: pathology and clinical sequelae. Postgrad. Med. 124, 90–97. 10.3810/pgm.2012.11.2615 23322142

[B25] Carvalho M. Silva B. M. Silva R. Valentao P. Andrade P. B. Bastos M. L. (2010). First report on *Cydonia oblonga* Miller anticancer potential: differential antiproliferative effect against human kidney and colon cancer cells. J. Agric. Food Chem. 58, 3366–3370. 10.1021/jf903836k 20192210

[B26] Chagas-Paula D. A. de-Oliveira R. B. da-Silva V. C. Gobbo-Neto L. Gasparoto T. H. Campanelli A. P.. (2011). Chlorogenic acids from *Tithonia diversifolia* demonstrate better anti-inflammatory effect than indomethacin and its sesquiterpene lactones. J. Ethnopharmacol. 136, 355–362. 10.1016/j.jep.2011.04.067 21575698

[B27] Chauhan P. S. Satti N. K. Sharma V. K. Dutt P. Suri K. A. Bani S. (2011). Amelioration of inflammatory responses by chlorogenic acid via suppression of pro-inflammatory mediators. J. Appl. Pharm. Sci. 1, 67–75.

[B28] Clark R. A. F. (1996). Wound repair: overview and general considerations, in The Molecular and Cellular Biology of Wound Repair, eds Clark R. A. F. Henson P. M (New York, NY: Plenum Press), 3–50.

[B29] Correa P. Haenszel W. Cuello C. Tannenbaum S. Archer M. (1975). A model for gastric cancer epidemiology. Lancet 2, 58–60. 10.1016/S0140-6736(75)90498-5 49653

[B30] Costa R. M. Magalhaes A. S. Pereira J. A. Andrade P. B. Valentao P. Carvalho M.. (2009). Evaluation of free radical-scavenging and anti-hemolytic activities of quince (*Cydonia oblonga*) leaf: a comparative study with green tea (*Camellia sinensis*). Food Chem. Toxicol. 47, 860–865. 10.1016/j.fct.2009.01.019 19271320

[B31] Daneshvand B. Ara K. M. Raofie F. (2012). Comparison of supercritical fluid extraction and ultrasound-assisted extraction of fatty acids from quince (*Cydonia oblonga* Miller) seed using response surface methodology and central composite design. J. Chromatogr.A 1252, 1–7. 10.1016/j.chroma.2012.06.063 22824221

[B32] De Benedictis F. M. Bush A. (2012). Corticosteroids in respiratory diseases in children. Am. J. Resp. Crit. Care Med. 185, 12–23. 10.1164/rccm.201107-1174CI 21920920

[B33] Della M. P. Lavagna A. Masoero G. Lombardo L. Crocella L. Pera A. (2002). Effectiveness of *Helicobacter pylori* eradication treatments in a primary care setting in Italy. Aliment. Pharmacol. Ther. 16, 1269–1275. 10.1046/j.1365-2036.2002.01244.x 12144576

[B34] De Tommasi N. De Simone F. Pizza C. Mahmood N. (1996). New tetracyclic sesterterpenes from *Cydonia vulgaris*. J. Nat. Prod. 59, 267–270. 10.1021/np9600513

[B35] Duke J. A. Bogenschutz-Godwin M. J. Ducelliar J. Duke P. A. K. (2002). Handbook of Medicinal Herbs, 2nd Edn. Boca Raton, FL: CRC Press.

[B36] Eddy A. A. (1996). Interstitial inflammation and fibrosis in rats with diet induced hypercholesterolemia. Kidney Int. 50, 1139–1149. 10.1038/ki.1996.421 8887271

[B37] Eichlerova I. Homolka L. Nerud F. (2006). Synthetic dye decolorization capacity of white rot fungus Dichomitus squalens. Bioresour. Technol. 97, 2153–2159. 10.1016/j.biortech.2005.09.014 16257199

[B38] Erdogan T. Gonenç T. Hortoglu Z. S. Demirci B. Başer K. H. C. Kıvçak B. (2012). Chemical composition of the essential oil of quince (*Cydonia Oblonga* Miller) leaves. Med. Aromat. Plants 1:134 10.4172/2167-0412.1000e134

[B39] Essafi-Benkhadir K. Refai A. Riahi I. Fattouch S. Karoui H. Essafi M. (2012). Quince (*Cydonia oblonga* Miller) peel polyphenols modulate LPS-induced inflammation in human THP-1-derived macrophages through NF-κB, p38MAPK and Akt inhibition. Biochem. Biophy. Res. Commun. 418, 180–185. 10.1016/j.bbrc.2012.01.003 22252293

[B40] Evans W. C. Evans D. Trease G. E. (2002). Trease and Evans pharmacognosy, 15th Edn. New York, NY: WB Saunders.

[B41] Fattouch S. Caboni P. Coroneo V. Tuberoso C. I. Angioni A. Dessi S.. (2007). Antimicrobial activity of Tunisian quince (*Cydonia oblonga* Miller) pulp and peel polyphenolic extracts. J. Agric. Food Chem. 55, 963–969. 10.1021/jf062614e 17263500

[B42] Fekri N. Khayami M. Heidari R. Jamee R. (2008). Chemical analysis of flaxseed, sweet basil, dragon head and quince seed mucilages. Res. J. Biol. Sci. 3, 166–170.

[B43] Ferguson L. R. (2001). Role of plant polyphenols in genomic stability. Res. Fundam. Mol. Mech. Mutagen. 475, 89–111. 10.1016/S0027-5107(01)00073-2 11295156

[B44] Ferreres F. Silva B. M. Andrade P. B. Seabra R. M. Ferreira M. A. (2003). Approach to the study of *C*-glycosyl flavones by ion trap HPLC-PAD-ESI/MS/MS: application to seeds of *Quince* (*Cydonia oblonga*). Phytochem. Anal. 14, 352–359. 10.1002/pca.727 14667061

[B45] Fischbach L. Evans E. L. (2007). Meta-analysis: the effect of antibiotic resistance status on the efficacy of triple and quadruple first-line therapies for *Helicbacter pylori*. Aliment. Pharmacol. Ther. 26, 343–357. 10.1111/j.1365-2036.2007.03386.x 17635369

[B46] Fleischmann P. Studer K. Winterhalter P. (2002). Partial purification and kinetic characterization of a carotenoid cleavage enzyme from *Quince* fruit (*Cydonia oblonga*). J. Agric. Food Chem. 50, 1677–1680. 10.1021/jf011184j 11879057

[B47] Ghafourian M. Tamri P. Hemmati A. (2015). Enhancement of human skin fibroblasts proliferation as a result of treating with quince seed mucilage. Jundishapur J. Nat. Pharm. Prod. 10, e18820–e18823. 10.17795/jjnpp-18820 25866719 PMC4379889

[B48] Ghanbari R. Anwar F. Alkharfy K. M. Gilani A. H. Saari N. (2012). Valuable nutrients and functional bioactives in different parts of olive (*Olea europaea* L) a review. Int. J. Mol. Sci. 13, 3291–3340. 10.3390/ijms13033291 22489153 PMC3317714

[B49] Gheisari H. R. Abhari K. H. (2014). Drying method effects on the antioxidant activity of quince (*Cydonia oblonga* Miller) tea. Acta Sci. Pol. Technol. Aliment. 13, 129–134. 10.17306/J.AFS.2014.2.2 24876308

[B50] Gholgholab H. (1961). Ghiah (in Farsi). Tehran: Tehran University Press.

[B51] Ghopur H. Usmanova S. K. Ayupbek A. Aisa H. A. (2012). A new chromone from seeds of Cydonia oblonga. Chem. Nat. Comp. 48, 562–564. 10.1007/s10600-012-0310-5

[B52] Gilani A. H. (1998). Novel developments from natural products in cardiovascular research. Phytother. Res. 12, 66–69.

[B53] Gilani A. H. Rahman A. U. (2005). Trends in ethnopharmacology. J. Ethnopharmacol. 100, 43–49. 10.1016/j.jep.2005.06.001 16127805

[B54] Girotti A. W. Thomas J. P. Jordan J. E. (1985). Lipid photooxidation in erythrocyte ghosts: sensitization of the membranes toward ascorbate-and superoxide-induced peroxidation and lysis. Arch. Biochem. Biophys. 236, 238–251. 10.1016/0003-9861(85)90623-X 2981506

[B55] Goldberg B. Stern A. (1977). The role of the superoxide anion as a toxic species in the erythrocyte. Arch. Biochem. Biophys. 178, 218–225. 10.1016/0003-9861(77)90187-4 189693

[B56] Graham D. Y. Shiotani A. (2008). New concepts of resistance in the treatment of *Helicobacter pylori* infections. Nat. Clin. Pract. Gastroenterol. Hepatol. 5, 321–331. 10.1038/ncpgasthep1138 18446147 PMC2841357

[B57] Griendling K. K. FitzGerald G. A. (2003). Oxidative stress and cardiovascular injury: part II: animal and human studies. Circulation 108, 2034–2040. 10.1161/01.CIR.0000093661.90582.c4 14581381

[B58] Grundemann C. Papagiannopoulos M. Lamy E. Sundermann V. M. Huber R. (2011). Immunomodulatory properties of a lemon-quince preparation (Gencydo^®^) as an indicator of anti-allergic potency. Phytomedicine 18, 760–768. 10.1016/j.phymed.2010.11.016 21256726

[B59] Halliwell B. Gutteridge J. M. (1985). The importance of free radicals and catalytic metal ions in human diseases. Mol. Aspects Med. 8, 89–193. 10.1016/0098-2997(85)90001-9 3908871

[B60] Halliwell B. Gutteridge J. M. (1986). Oxygen free radicals and iron in relation to biology and medicine: some problems and concepts. Arch. Biochem. Biophys. 246, 501–514. 10.1016/0003-9861(86)90305-X 3010861

[B61] Hamauzu Y. Irie M. Kondo M. Fujita T. (2008). Anti-ulcerative properties of crude polyphenols and juice of apple and Chinese quince extracts. Food Chem. 108, 488–495. 10.1016/j.foodchem.2007.10.084 26059126

[B62] Hamid A. Mehdi R. Shahrzad N. S. Ajalli M. (2014). Synergistic antifungal effects of quince leaf's extracts and silver nanoparticles on Aspergillus niger. J. Appl. Biol. Sci. 8, 10–13.

[B63] Hamid H. F. Moezzi A. Khouzani M. A. MahmoudJanlou Y. Niknejad F. Faramarzi M. A. (2013). Synthetic dye decolorization by three sources of fungal laccase. Res. J. Chem. Environ. 17, 76–81. 10.1186/1735-2746-9-27 PMC3564790 23369690

[B64] Hansson G. K. (2005). Inflammation, atherosclerosis, and coronary artery disease. N. Engl. J. Med. 352, 1685–1695. 10.1056/NEJMra043430 15843671

[B65] Heep M. Kist M. Strobel S. Beck D. Lehn N. (2000). Secondary resistance among 554 isolates of *Helico-bacter pylori* after failure of therapy. Eur. J. Clin. Microbiol. Infect. Dis. 19, 538–541. 10.1007/s100960000288 10968325

[B66] Hemmati A. A. Mohammadian F. (2000). An investigation into the effects of mucilage of quince seeds on wound healing in rabbit. J. Herbs Sp. Med. Plants 7, 41–46. 10.1300/J044v07n04_05

[B67] Hopur H. Asrorov A. M. Qingling M. Yili A. Ayupbek A. Nannan P. (2011). HPLC Analysis of polysaccharides in *Quince* (*Cydonia Oblonga* Mill. var. *maliformis*) fruit and PTP1B inhibitory activity. Nat. Prod. J. 1, 146–150. 10.2174/2210315511101020146

[B68] Huber R. Stintzing F. C. Briemle D. Beckmann C. Meyer U. Grundemann C. (2012). *In-vitro* anti-allergic effects of aqueous fermented preparations from Citrus and Cydonia fruits. Planta Med. 78, 334–340. 10.1055/s-0031-1280455 22193979

[B69] Husain Q. Jan U. (2000). Detoxification of phenols and aromatic amines from polluted waste water by using phenol oxidases. J. Sci. Ind. Res. 59, 286–293.

[B70] Huxley A. Griffiths M. Levy M. (eds.). (1999). The New RHS Dictionary of Gardening. London: Grove's Dictionaries. Paper and slipcase Edn.

[B71] Irshaid F. Mansi K. Bani-Khaled A. Aburjia T. (2012). Hepatoprotetive, cardioprotective and nephroprotective actions of essential oil extract of *Artemisia sieberi* in alloxan induced diabetic rats. Iran. J. Pharm. Res. 11, 1227–1234. 24250557 PMC3813169

[B72] Ishihara M. Tsuneya T. Shiota H. Shiga M. Nakatsu K. (1986). Identification of new constituents of quince fruit flavor (*Cydonia oblonga* Mill. = C. vulgaris Pers). J. Org. Chem. 51, 491–495. 10.1021/jo00354a016

[B73] Jackson S. P. Bartek J. (2009). The DNA-damage response in human biology and disease. Nature 46, 1071–1078. 10.1038/nature08467 19847258 PMC2906700

[B74] Jaladat A. M. Atarzadeh F. Rezaeizadeh H. Mofid B. Mosalaie A. Farhan F.. (2015). Botanicals: an alternative remedy to radiotherapy-induced dysuria. Complement Ther. Med. 23, 90–99. 10.1016/j.ctim.2014.11.004 25637157

[B75] Janbaz K. Shabbir A. Mehmood M. H. Gilani A. H. (2013). Insight into mechanism underlying the medicinal use of *Cydonia oblonga* in gut and airways disorders. J. Animal Plant Sci. 23, 330–336.

[B76] Joles J. A. Kunter U. T. A. Janssen U. L. F. Kriz W. Rabelink T. J. Koomans H. A.. (2000). Early mechanisms of renal injury in hypercholesterolemic or hypertriglyceridemic rats. J. Am. Soc. Nephrol. 11, 669–683. 10752526 10.1681/ASN.V114669

[B77] Jones R. Mann T. Sherins R. (1979). Peroxidative breakdown of phospholipids in human spermatozoa, spermicidal properties of fatty acid peroxides, and protective action of seminal plasma. Fertil. Steril. 31, 531–537. 10.1016/S0015-0282(16)43999-3 446777

[B78] Jouki M. Yazdi F. T. Mortazavi S. A. Koocheki A. (2014). Quince seed mucilage films incorporated with oregano essential oil: physical, thermal, barrier, antioxidant and antibacterial properties. Food Hydrocolloids 36, 9–19. 10.1016/j.foodhyd.2013.08.030

[B79] Jouyban A. Shoja M. M. Ardalan M. R. Khoubnasabjafari M. Sadighi A. Tubbs R. S. (2011). The effect of quince leaf decoction on renal injury induced by hypercholesterolemia in rabbits: a pilot study. J. Med. Plants Res. 5, 5291–5295.

[B80] Karar M. G. E. Pletzer D. Jaiswal R. Weingart H. Kuhnert N. (2013). Identification, characterization, isolation and activity against *Escherichia coli* of *Quince* (*Cydonia oblonga*) fruit polyphenols. Food Res. Int. 65, 121–129. 10.1016/j.foodres.2013.10.040

[B81] Kasiske B. L. O'Donnell M. P. Garvis W. J. Keane W. F. (1988). Pharmacologic treatment of hyperlipidemia reduces glomerular injury in rat 5/6 nephrectomy model of chronic renal failure. Circ. Res. 62, 367–374. 3338121 10.1161/01.res.62.2.367

[B82] Kawahara T. Lizuka T. (2011). Inhibitory effect of hot-water extract of quince (*Cydonia oblonga*) on immunoglobulin E-dependent late-phase immune reactions of mast cells. Cytotechnology 63, 143–152. 10.1007/s10616-010-9323-8 21264509 PMC3080480

[B83] Khademi F. Danesh B. Nejad M. D. Rad J. S. (2013). The comparative effects of atorvastatin and quince leaf extract on atherosclerosis. Iran. Red Cres. Med. J. 15, 639–643. 10.5812/ircmj.4030 24578828 PMC3918185

[B84] Khoubnasabjafari M. Jouyban A. (2011). A review of phytochemistry and bioactivity of quince (*Cydonia oblonga* Mill). J. Med. Plants Res. 5, 3577–3594.

[B85] Kirimer N. Tunalier Z. Can B. K. H. Cingi I. (1997). Antispasmodic and spasmogenic effects of *Scolymus hispanicus* and taraxasteryl acetate on isolated ileum preparations. Planta Med. 63, 556–558. 10.1055/s-2006-957765 9434612

[B86] Kobayashi T. Itabe H. Inoue K. Nojima S. (1985). Peroxidation of liposomes in the presence of human erythrocytes and induction of membrane damage of erythrocytes by peroxidized liposomes. Biomembranes 814, 170–178. 10.1016/0005-2736(85)90433-X 4038885

[B87] Krishnaswamy K. (2008). Traditional Indian spices and their health. Asian Pac. J. Clin. Nutr. 17, 265–268. 18296352

[B88] Kusari S. Singh S. Jayabaskaran C. (2014). Re-thinking production of Taxol® (paclitaxel) using endophyte biotechnology. Trends Biotechnol. 32, 304–311. 10.1016/j.tibtech.2014.03.011 24810040

[B89] Labat-Robert J. Robert L. (2014). Longevity and aging. role of free radicals and xanthine oxidase. a review. Pathol. Biol. 62, 61–66. 10.1016/j.patbio.2014.02.009 24650523

[B90] Lattanzio V. Kroon P. A. Linsalata V. Cardinali A. (2009). Globe artichoke: a functional food and source of nutraceutical ingredients. J. Func. Foods 1, 131–144. 10.1016/j.jff.2009.01.002

[B91] Lindberg B. Mosihuzzaman M. Nahar N. Abeysekera R. M. Brown R. G. Willison J. H. M. (1990). An unusual (4-O-methyl-O-glucurono)-O-xylan isolated from the mucilage of seeds of the quince tree (*Cydonia oblonga*). Carbohyd. Res. 207, 307–310. 10.1016/0008-6215(90)84057-2

[B92] Lorenz P. Berger M. Bertrams J. Wende K. Wenzel K. Lindequist U.. (2008). Natural wax constituents of a supercritical fluid CO_2_ extract from Quince (*Cydonia oblonga* Mill) pomace. Anal. Bioanal. Chem. 391, 633–646. 10.1007/s00216-008-2000-5 18418588

[B93] Lutz-Roder A. Schneider M. Winterhalter P. (2002). Isolation of two new ionone glucosides from quince (*Cydonia oblonga* Miller) leaves. Nat. Prod. Lett. 16, 119–122. 10.1080/10575630290020028 11990428

[B94] Madamanchi N. R. Vendrov A. Runge M. S. (2004). Oxidative stress and vascular disease. Arterioscler. Thromb. Vasc. Biol. 25, 29–38. 10.1161/01.ATV.0000150649.39934.13 15539615

[B95] Magalhaes A. S. Siva B. M. Pereira J. A. Andrade P. B. Valentao P. Carvalho M. (2009). Protective effect of Quince (*Cydonia oblonga* Miller) fruit against oxidative hemolysis of human erythrocytes. Food Chem. Toxicol. 47, 1372–1377. 10.1016/j.fct.2009.03.017 19306906

[B96] Marwat S. K. Khan M. A. Khan M. A. Ahmad M. Zafar M. Fazal-ur-rehman Sultana S. (2009). Fruit plant species mentioned in the Holy Qura'n and Ahadith and their ethnomedicinal importance. Am. Eurasian J. Agric. Environ. Sci. 5, 284–295.

[B97] McGowan C. C. Cover T. L. Blaser M. J. (1996). *Helicobacter pylori* and gastric acid: biological and therapeutic implications. Gastroenterology 110, 926–938. 10.1053/gast.1996.v110.pm8608904 8608904

[B98] McQuaid K. R. (2007). Drugs used in the treatment of gastrointestinal disease, in Basic and Clinical Pharmacology, 10th Edn., ed Katzung B. G. (New York, NY: McGraw Hill Companies), 1029–1035.

[B99] Middleton E. Kandaswami C. (1992). Effects of flavonoids on immune and inflammatory cell functions. Biochem. Pharmacol. 43, 1167–1179. 10.1016/0006-2952(92)90489-6 1562270

[B100] Middleton E. Kandaswami C. Theoharides T. C. (2000). The effects of plant flavonoids on mammalian cells: implications for inflammation, heart disease, and cancer. Pharmacol. Rev. 52, 673–751. 11121513

[B101] Minaiyan M. Ghannadi A. Etemad M. Mahzouni P. (2012). A study of the effects of *Cydonia oblonga* Miller (*Quince*) on TNBS-induced ulcerative colitis in rats. Res. Pharm. Sci. 7, 103–110. 23181087 PMC3501898

[B102] Mirmohammadlu M. Hosseini S. H. Kamalinejad M. Gavgani M. E. Noubarani M. Eskandari M. R. (2015). Hypolipidemic, hepatoprotective and renoprotective effects of *Cydonia oblonga* Mill. fruit in streptozotocin-induced diabetic rats. Iran. J. Pharm. Res. 14, 1207–1214. 26664388 PMC4673949

[B103] Mobarakeh K. M. Etebari M. Zolfaghari B. Dehkordi A. J. (2015). Evaluation of genoprotective effects of hydroalcoholic and polyphenolic extracts of *Quince* by comet assay. J. Rep. Pharm. Sci. 4, 141–147.

[B104] Moore K. J. Tabas I. (2011). Macrophages in the pathogenesis of atherosclerosis. Cell 145, 341–355. 10.1016/j.cell.2011.04.005 21529710 PMC3111065

[B105] Mueller C. F. Laude K. McNally J. S. Harrison D. G. (2005). ATVB in focus: redox mechanisms in blood vessels. Arterioscler. Thromb. Vasc. Biol. 25, 274–278. 10.1161/01.ATV.0000149143.04821.eb 15514203

[B106] Muhammad G. Hussain M. A. Anwar F. Ashraf M. Gilani A. H. (2014). *Alhagi*: a plant genus rich in bioactives for pharmaceuticals. Phytother. Res. 29, 1–13. 10.1002/ptr.5222 25256791

[B107] Muhammad G. Hussain M. A. Jantan I. Bukhari S. N. A. (2016). *Mimosa pudica* L., a high-value medicinal plant as a source of bioactives for pharmaceuticals. Compr. Rev. Food Sci. Food Saf. 15, 303–315. 10.1111/1541-4337.12184 33371596

[B108] Mune M. Meydani M. Gong J. Fotouhi N. Ohtani H. Smith D.. (1999). Effect of dietary fish oil, vitamin E, and probucol on renal injury in the rat. J. Nutr. Biochem. 10, 539–546. 10.1016/S0955-2863(99)00042-X 15539334

[B109] Murcia M. A. Jimenez A. M. Martinez-Tome M. (2001). Evaluation of the antioxidant properties of Mediterranean and tropical fruits compared with common food additives. J. Food Prot. 64, 2037–2046. 11770635 10.4315/0362-028x-64.12.2037

[B110] Nadkarni K. M. (1976). Indian Materia Medica with Ayurvedic, Unani-tibbi, Siddha, Allopathic, Homeopathic, Naturopathic & Home Remedies, Appendices & Indexes, 3rd Edn. Bombay: Popular Prakashan.

[B111] Nandi B. K. Goswami A. Purkait M. K. (2009). Adsorption characteristics of brilliant green dye on kaolin. J. Hazard. Mat. 161, 387–395. 10.1016/j.jhazmat.2008.03.110 18456401

[B112] Ndip R. N. Tarkang A. E. Echakachi C. M. Malongue A. Akoachere J. F. Ndip L. M.. (2007a). *. In vitro* antimicrobial activity of selected honeys on clinical isolates of Helicobacter pylori. Afr. Health Sci. 7, 228–231. 21499488 PMC3074369

[B113] Ndip R. N. Tarkang A. E. M. Mbullah S. M. Luma H. N. Malongue A. Ndip L. M.. (2007b). *In vitro* anti-*Helicobacter pylori* activity of extracts of selected medicinal plants from North West Cameroon. J. Ethnopharmacol. 114, 452–457. 10.1016/j.jep.2007.08.037 17913416

[B114] Nijveldt R. J. Van-Nood E. Van-Hoorn D. E. Boelens P. G. Van-Norren K. Van-Leeuwen P. A. (2001). Flavonoids: a review of probable mechanisms of action and potential applications. Am. J. Clin. Nutr. 74, 418–425. 11566638 10.1093/ajcn/74.4.418

[B115] Niki E. Komuro E. Takahashi M. Urano S. Ito E. Terao K. (1988). Oxidative hemolysis of erythrocytes and its inhibition by free radical scavengers. J. Biol. Chem. 263, 19809–19814. 3198651

[B116] Oliveira A. P. Pereira J. A. Andrade P. B. Valentao P. Seabra R. M. Silva B. M. (2007). Phenolic profile of *Cydonia oblonga* Miller leaves, J. Agric. Food Chem. 55, 7926–7930. 10.1021/jf0711237 17711340

[B117] Oliveira A. P. Pereira J. A. Andrade P. B. Valentao P. Seabra R. M. Silva B. M. (2008). Organic acid composition of *Cydonia oblonga* Miller leaf. Food Chem. 111, 393–399. 10.1016/j.foodchem.2008.04.004 26047441

[B118] Osman A. G. Koutb M. Sayed A. E. D. H. (2010). Use of hematological parameters to assess the efficiency of quince (*Cydonia oblonga* Miller) leaf extract in alleviation of the effect of ultraviolet-A radiation on African catfish *Clarias gariepinus* (Burchell, 1822). J. Photochem. Photobiol. B. 99, 1–8. 10.1016/j.jphotobiol.2010.01.002 20206545

[B119] Pacifico S. Gallicchio M. Fiorentino A. Fischer A. Meyer U. Stintzing F. C. (2012). Antioxidant properties and cytotoxic effects on human cancer cell lines of aqueous fermented and lipophilic quince (*Cydonia oblonga* Mill) preparations. Food Chem. Toxicol. 50, 4130–4135. 10.1016/j.fct.2012.07.061 23034449

[B120] Padron R. S. Mas J. Zamora R. Riverol F. Licea M. Mallea L.. (1989). Lipids and testicular function. Int. Urol. Nephrol. 21, 515–519. 10.1007/BF02549590 2613482

[B121] Papp N. Szabo T. Szabo Z. Nyeki J. Stefanovits-Banyai E. I. Hegedus A. (2013). Antioxidant capacity and total polyphenolic content in quince (*Cydonia oblonga* Mill) fruit. Int. J. Hort. Sci. 19, 33–36.

[B122] Pashkow F. J. (2011). Oxidative stress and inflammation in heart disease: do antioxidants have a role in treatment and/or prevention. Int. J. Inflam. 2011:514623. 10.4061/2011/514623 21860805 PMC3157078

[B123] Patel P. N. Patel H. S. (2012). Removal and decolorization of dye bearing textile effluents by sulfinated furfural-acetone resin. Adv. Appl. Sci. Res. 3, 2693–2699.

[B124] Pengthamkeerati P. Satapanajaru T. Chatsatapattayakul N. Chairattanamanokorn P. Sananwai N. (2010). Alkaline treatment of biomass fly ash for reactive dye removal from aqueous solution. Desalination 261, 34–40. 10.1016/j.desal.2010.05.050

[B125] Perry S. Sanchez M. L. Yang S. Haggerty T. D. Hurst P. Perez-Perez G.. (2006). Gastroenteritis and transmission of *Helicobacter pylori* infection in house-holds. Emerg. Infect. Dis. 12, 1701–1708. 10.3201/eid1211.060086 17283620 PMC3372328

[B126] Poredos P. Jezovnik M. K. (2011). Dyslipidemia, statins, and venous thromboembolism. in Semin Thromb. Hemost. 37, 897–902. 10.1055/s-0031-1297368 22198854

[B127] Prajapati N. D. Purohit S. S. Sharma A. K. Kumar T. (2006). A Handbook of Medicinal Plants. Jodhpur: Agrobios, Section II, 86.

[B128] Qasim A. O'Morain C. A. (2002). Review article: treatment of *Helicobacter pylori* infection and factors influencing eradication. Aliment Pharmacol. Ther. 16, 24–30. 10.1046/j.1365-2036.2002.0160s1024.x 11849124

[B129] Rahmatullah M. Ferdausi D. Mollik M. A. H. Jahan R. Chowdhury M. H. Haque W. M. (2010a). A survey of medicinal plants used by Kaverajes of Chalna area, Khulna District, Bangladesh. Afr. J. Trad. Complement Alt. Med. 7, 91–97. 21304618 10.4314/ajtcam.v7i2.50859 PMC3021158

[B130] Rahmatullah M. Kabir A. A. B. T. Rahman M. Hossan S. Khatun Z. Khatun A. (2010b). Ethnomedicinal practices among a minority group of christians residing in Mirzapur village of Dinajpur District, Bangladesh. Adv. Nat. Appl. Sci. 4, 45–51.

[B131] Ramirez-Torres M. A. Carrera A. Zambrana M. (2000). High incidence of hyperestrogenemia and dyslipidemia in a group of infertile men. Ginecol. Obstet. Mex. 68, 224–229. 10902292

[B132] Rocha M. Apostolova N. Hernandez-Mijares A. Herance R. Victor V. M. (2010). Oxidative stress and endothelial dysfunction in cardiovascular disease: mitochondria-targeted therapeutics. Curr. Med. Chem. 17, 3827–3841. 10.2174/092986710793205444 20858217

[B133] Roediger W. E. (2010). The starved colon diminished mucosal nutrition, diminished absorption, and colitis. Dis. Colon Rect. 33, 858–862. 10.1007/BF02051922 2209275

[B134] Rolandelli R. H. Saul S. H. Settle R. G. Jacobs D. O. Trerotola S. O. Rombeau J. L. (1988). Comparison of parenteral nutrition and enteral feeding with pectin in experimental colitis in the rat. Am. J. Clin. Nutr. 47, 715–721. 3128101 10.1093/ajcn/47.4.715

[B135] Rop O. Balik J. Reznicek V. Jurikova T. Skardova P. Salas P. (2011). Chemical characteristics of fruits of some selected quince (*Cydonia oblonga* Miller) cultivars. Cazech J. Food Sci. 29, 65–73.

[B136] Rubin R. Strayer D. S. Rubin E. (eds.). (2012). Rubin's Pathology: Clinicopathologic Foundations of Medicine, 6th Edn. Lippincott Williams and Wilkins.

[B137] Russell W. Duthie G. (2011). Plant secondary metabolites and gut health: the case for phenolic acids. Proc. Nutr. Soc. 70, 389–396. 10.1017/S0029665111000152 21781364

[B138] Sato Y. Itagaki S. Kurokawa T. Ogura J. Kobayashi M. Hirano T.. (2011). *. In-vitro* and *In-vivo* antioxidant properties of chlorogenic acid and caffeic acid. Int. J. Pharm. 403, 136–138. 10.1016/j.ijpharm.2010.09.035 20933071

[B139] Sezik E. Yesilada E. Hondo G. Takaishi Y. Takeda Y. Tanaka T. (2001). Traditional medicine in Turkey X. Folk medicine in Central Anatolia. J. Ethnopharamacol. 75, 95–115. 10.1016/S0378-8741(00)00399-8 11297840

[B140] Sharma R. Joshi V. K. Rana J. C. (2011). Nutritional composition and processed products of *Quince* (*Cydonia oblonga* Mill). Indian J. Nat. Prod. Res. 2, 354–357.

[B141] Shinomiya F. Hamauzu Y. Kawahara T. (2009). Anti-allergic effect of a hot-water extract of quince (*Cydonia oblonga*). Biosci. Biotechnol. Biochem. 73, 1773–1778. 10.1271/bbb.90130 19661701

[B142] Silva B. M. Andrade P. B. Ferreres F. Seabra M. R. Oliveira M. B. P. P. Margarida A. F. (2005). Composition of *Quince* (*Cydonia oblonga* Miller) seeds: phenolics, organic acids and free amino acids. Nat. Prod. Res. 19, 275–281. 10.1080/14786410410001714678 15702641

[B143] Silva B. M. Andrade P. B. Goncalves A. C. Seabra R. M. Oliveria M. B. Ferreira M. A. (2004b). Influence of jam processing upon the contents of phenolics, organic acids and free amino acids in quince fruit (*Cydonia oblonga* Miller). Eur. Food Res. Technol. 218, 385–389. 10.1007/s00217-003-0845-6

[B144] Silva B. M. Andrade P. B. Mendes G. C. Seabra R. M. Ferreira M. A. (2002a). Phenolic profile of quince fruit (*Cydonia oblonga* Miller) (Pulp and Peel). J. Agric. Food Chem. 50, 4615–4618. 10.1021/jf0203139 12137485

[B145] Silva B. M. Andrade P. B. Mendes G. C. Seabra R. M. Ferreira M. A. (2002b). Study of the organic acids composition of *Quince* (*Cydonia oblonga* Miller) fruit and jam. J. Agric. Food Chem. 50, 2313–2317. 10.1021/jf011286+ 11929290

[B146] Silva B. M. Andrade P. B. Valentao P. Ferreres F. Seabra R. M. Ferreira M. A. (2004a). *Quince* (*Cydonia oblonga*). Miller) fruit (pulp, peel, and seed) and jam: antioxidant activity. J. Agric. Food Chem. 52, 4705–4712. 10.1021/jf040057v 15264903

[B147] Silva B. M. Casal S. Andrade P. B. Seabra M. R. Oliveira M. B. P. P. Ferreira M. A. (2004c). Free amino acid composition of *Quince* (*Cydonia oblonga* Miller) fruit (pulp and peel) and jam. J. Agric. Food Chem. 52, 1201–1206. 10.1021/jf030564x 14995121

[B148] Slpponen P. Kekki M. Haapakoski J. Ihamaki T. Siurala M. (1985). Gastric cancer risk in chronic atrophic gastritis: statistical calculations of cross-sectional data. Int. J. Cancer 35, 173–177. 10.1002/ijc.2910350206 3871738

[B149] Smith F. Montgomery R. (1959). The Chemistry of Plant Gums and Mucilages. New York, NY: Reinhold Pub. Co.

[B150] Stenstrom B. Mendis A. Marshall B. (2008). *Helicobacter pylori-*the latest in diagnosis treatment. Aus. Fam. Phys. 37, 608–612. 18704207

[B151] Stortelers C. Kerkhoven R. Moolenaar W. H. (2008). Multiple actions of lysophosphatidic acid on fibroblasts revealed by transcriptional profiling. BMC Genomics 9:387. 10.1186/1471-2164-9-387 18702810 PMC2536681

[B152] Szychowski P. J. Munera-Picazo S. Szumny A. Carbonell-Barrachina A. A. Hernandez F. (2014). Quality parameters, bio-compounds, antioxidant activity and sensory attributes of Spanish quinces (*Cydonia oblonga* Miller). Sci. Horti. 165, 163–170. 10.1016/j.scienta.2013.11.028

[B153] Tabata M. Honda G. Sezik E. Yesilada E. (1993). Eds., A Report on Traditional Medicine and Medicinal Plants in Turkey (1990, 1991). Japan: Kyoto University Press.

[B154] Tajoddini A. Rafieian-Kopaei M. Namjoo A. R. Sedeh M. Ansari R. Shahinfard N. (2013). Effect of ethanolic extract of *Cydonia oblonga* seed on the healing of second-degree burn wounds. Armaghan Danesh 17, 494–501.

[B155] Tamri P. Hemmati A. Borouierdnia M. G. (2014). Wound healing properties of quince seed mucilage: *in vivo* evaluation in rabbit full-thickness wound model. Int. J. Surg. 12, 843–847. 10.1016/j.ijsu.2014.06.016 25017948

[B156] Tateo F. Bononi M. (2010). Headspace-SPME analysis of volatiles from quince whole fruit. J. Essen. Oil Res. 22, 416–418. 10.1080/10412905.2010.9700360

[B157] Torkelson A. R. (1995). The Cross Name Index to Medicinal Plants. London: CRC Press.

[B158] Trovato A. Taviano M. F. Pergolizzi S. Campolo L. De Pasquale R. Miceli N. (2010). Citrus bergamia risso and Poiteau juice protects against renal injury of diet-induced hypercholesterolemia in rats. Phytother. Res. 24, 514–519. 10.1002/ptr.2971 19655295

[B159] Tsunevay T. Ishihara M. Shiota H. Shiga M. (1983). Volatile components of *Quince* fruit (*Cydonia oblonga* Mill). Agric. Biol. Chem. 47, 2495–2502. 10.1271/bbb1961.47.2495

[B160] Tuzlaci E. Tolon E. (2000). Turkish folk medicinal plants, part III: sile (Istanbul). Fitoterapia 71, 673–685. 10.1016/S0367-326X(00)00234-3 11077175

[B161] Umano K. Shoji A. Hagi Y. Shibamoto T. (1986). Volatile constituents of peel of quince fruit, *Cydonia oblonga* Miller. *J. Agric*. Food Chem. 34, 593–596. 10.1021/jf00070a003

[B162] Usmanghani K. Saeed A. Alam M. T. (1997). Indusyunic Medicine. Karachi:University of Karachi Press.

[B163] Vaez H. Hamidi S. Arami S. (2014). Potential of *Cydonia oblonga* leaves in cardiovascular disease. Hypothesis 12, 1–10. 10.5779/hypothesis.v12i1.356

[B164] Vignon M. R. Gey C. (1998). Isolation, ^1^H and ^13^C NMR studies of (4-*O*-methyl-d-glucurono)-d-xylans from luffa fruit fibres, jute bast fibres and mucilage of quince tree seeds. Carbohydr. Res. 307, 107–111. 10.1016/S0008-6215(98)00002-0

[B165] Wang X. Jia W. Zhao A. (2006). Anti-influenza agents from plants and traditional Chinese medicine. Phytother. Res. 20, 335–341. 10.1002/ptr.1892 16619359

[B166] Winter C. A. Risley E. A. Nuss G. W. (1962). Carrageenin induced edema in hind paw of the rat as an assay for anti-inflammatory drugs. Exp. Biol. Med. 111, 544–547. 10.3181/00379727-111-27849 14001233

[B167] Winterhalter P. Schreier P. (1988). Free and bound C_13_ norisoprenoids in *Quince* (*Cydonia oblonga*, Mill) fruit. J. Agric. Food Chem. 36, 1251–1256. 10.1021/jf00084a031

[B168] World Health Organization (WHO) (2002). The World Health Report: Reducing Risks, Promoting Healthy life. Geneva.

[B169] Wojdylo A. Oszmianski J. Bielicki P. (2013). Polyphenolic composition, antioxidant activity, and polyphenol oxidase (PPO) activity of *Quince* (*Cydonia oblonga* Miller) varieties. J. Agric. Food Chem. 61, 2762–2772. 10.1021/jf304969b 23461298

[B170] Xavier R. J. Podolsky D. K. (2007). Unravelling the pathogenesis of inflammatory bowel disease. Nature 448, 427–434. 10.1038/nature06005 17653185

[B171] Yildirim A. Oktay M. Bilaloglu V. (2001). The antioxidant activity of the leaves of Cydonia vulgaris. Turk. J. Med. Sci. 31, 23–27.

[B172] Zhou W. Abdurahman A. Umar A. Iskander G. Abdusalam E. Berke B.. (2014). Effects of *Cydonia oblonga* Miller extracts on blood hemostasis, coagulation and fibrinolysis in mice, and experimental thrombosis in rats. J. Ethnopharmacol. 154, 163–169. 10.1016/j.jep.2014.03.056 24704668

[B173] Zohalinezhad M. E. Imanieh M. H. Samani S. M. Mohagheghzadeh A. Dehghani S. M. Haghighat M.. (2015). Effects of Quince syrup on clinical symptoms of children with symptomatic gastroesophageal reflux disease: a double-blind randomized controlled clinical trial. Compl. Ther. Clin. Pract. 21, 268–276. 10.1016/j.ctcp.2015.09.005 26573454

[B174] Zsivanovits G. Szigeti F. Mohacsi-Farkas C. (2013). Investigation of antimicrobial inhibition effect of quince fruit extract by rapid impedance method, in International Scientific-Practical Conference, Food, Technology and Health (Plovdiv), 264–270.

